# Organic Solvents-Based Offline Aerosol Mass Spectrometry (SOff-AMS) for Comprehensive Chemical Characterization of Ambient Organic Aerosol

**DOI:** 10.1021/acs.est.5c08949

**Published:** 2025-08-19

**Authors:** Peeyush Khare, Abdul Aziz Kurdieh, Yufang Hao, Manousos-Ioannis Manousakas, Lubna Dada, Anna Tobler, Kristty Schneider-Beltran, Evangelia Diapouli, Alicja Skiba, Katarzyna Styszko, Jay G. Slowik, André S. H. Prévôt, Kaspar R. Daellenbach

**Affiliations:** † PSI Center for Energy and Environmental Sciences, 28498 Paul Scherrer Institute, 5232 Villigen, Switzerland; ‡ ENRACT, Institute of Nuclear & Radiological Sciences and Technology, Energy & Safety, N.C.S.R. “Demokritos”, Agia Paraskevi 15310, Greece; § AGH University of Krakow, Faculty of Physics and Applied Computer Science, 30-059 Krakow, Poland; ∥ AGH University of Krakow, Faculty of Energy and Fuels, 30-059 Krakow, Poland

**Keywords:** solvents, offline aerosol mass spectrometry, atmospheric aerosols, source apportionment

## Abstract

The chemical composition of atmospheric aerosols is frequently investigated offline via mass spectrometry techniques. The established offline aerosol mass spectrometry (Off-AMS) uses water for extracting airborne particulate filter samples, efficiently detecting water-soluble constituents of organic aerosols (OA), which are prevalent in secondary OA (SOA), oxygenated primary OA (POA), and aged POA. Consequently, sources with substantial water-insoluble fraction may be undetectable via Off-AMS or be subject to increased uncertainties due to the absence of useful markers (e.g., polycyclic aromatic and aliphatic hydrocarbons). This potentially compromises the investigation of primary and less-aged OA from diverse anthropogenic sources (e.g., traffic, coal combustion, waste burning, tire wear, among others). Here, we present a new analytical method that combines Off-AMS with organic solvent-based sample extraction (termed: SOff-AMS) to extract and quantify both aged and fresh aerosols simultaneously. Ultrahigh-purity methanol and high-purity acetone were used, alongside water as a reference, and the extracts were reaerosolized to be analyzed via a high-resolution time-of-flight aerosol mass spectrometer (ToF-AMS). Multiseason airborne particulate matter (PM) filter samples collected in urban and rural environments were used in these tests. The organic solvents extracted substantially higher fractions of organic carbon, which for winter samples ranged from 45 to 85% of the total organic carbon in comparison to 12–40% in water alone. The AMS spectra of samples extracted in organic solvents showed significantly increased contributions from OA fragments that are known tracers of fresh and aged emissions. These included small and polycyclic aromatic hydrocarbons and oxygenated and reduced nitrogen-containing fragments that were enhanced over a broad range of factors (1.2–50). A comparison with an online quadrupole aerosol chemical speciation mass spectrometer (Q-ACSM) in Krakow showed highly similar spectra, demonstrating that SOff-AMS-based offline measurements can provide very similar information as the online data. Future SOff-AMS-based source apportionment could identify air pollution sources more comprehensively regardless of sampling locations, particle sizes, and seasonal conditions, especially in complex urban areas with both primary and secondary source contributors.

## Graphical abstract

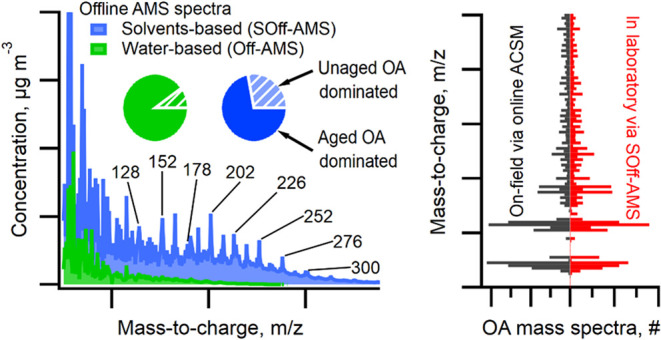

## 1 Introduction

Organic aerosols (OA) constitute a large fraction of airborne particulate matter and impact both the climate and human health.^1,2^ OA is either directly emitted (primary; POA) or is formed via atmospheric oxidation of gas-phase precursors (secondary; SOA) emitted from diverse anthropogenic (e.g., motor vehicles, solid-fuel combustion, asphalt) and biogenic (e.g., forests) sources. It is essential to characterize the chemical composition of OA in order to identify its contributing sources and constrain their impact.

In recent decades, advances in mass spectrometry have significantly enhanced our understanding of the OA composition and formation processes.^3–5^ Online techniques, e.g., time-of-flight aerosol mass spectrometry (ToF-AMS) and extractive electrospray ionization (EESI)-ToF MS, provide OA composition with very high time-resolution at both bulk and molecular levels for nontrace ambient concentrations.^6–8^ Other online mass spectrometers can also characterize low ambient OA concentrations at longer time intervals (e.g., FIGAERO–CIMS).^9^ Despite these benefits, the application of online mass spectrometers for long-term air quality studies is severely restricted by high logistical and maintenance costs in many resource-limited urban and rural locations around the world.

Offline techniques (e.g., liquid chromatography (LC)-ToF, LC-Orbitrap, laser desorption ionization-ToF, two-dimensional gas chromatography-ToF, FIGAERO-EESI-MS, among others) overcome these issues by measuring the composition of ambient OA sampled on quartz or Teflon filters in a laboratory.^10–17^ The samples can be collected using high or low volume samplers that can be easily transported and installed in different locations. Offline methods considerably expand the regions where OA composition can be investigated cost-effectively via state-of-the-art mass spectrometry. Filter sample extracts in organic solvents (e.g., methanol, acetone, acetonitrile) are mostly analyzed via soft-ionization-based analytical techniques. However, the detailed chemical information obtained from soft-ionization-based, nontargeted offline analyses is often difficult to quantify due to measurement uncertainties originating from sample preparation, relative differences in analyte sensitivity to ionization schemes, and the unavailability of calibration standards.

The more recently developed offline-AMS (Off-AMS) technique has permitted quantifiable measurements of ambient OA in laboratory conditions.^18–21^ These measurements can be applied to source apportionment algorithms (e.g., positive matrix factorization) to quantify the contributions of OA sources. However, Off-AMS typically uses Milli-Q water for sample extraction, and filters extracted in organic solvents have been rarely measured via AMS.^22,23^ This limits the analysis to the water-soluble fraction of OA (WSOA) that is largely constituted by SOA. Depending on the environment, sources, and seasons, WSOA can range from 15 to 80% of ambient OA.^24–27^ It is often low during winter in regions with complex emission portfolios dominated by combustion-based sources, e.g., motor vehicles and solid-fuel burning, where WSOA may constitute less than 50% of the total OA mass. Consequently, source apportionment analyses based on Off-AMS measurements can mainly identify sources that are fully or at least somewhat water-soluble. The sources of POA and non-WSOA must be estimated either via empirical methods or corrected for with substantial uncertainties. Such corrections can introduce additional uncertainties in analyses and source apportionment outcomes.^21,28,29^

Here, we present a new analytical approach to overcome these challenges by coupling Off-AMS with filter samples extracted in organic solvents (hereon SOff-AMS). Via analyzing ambient samples collected from different environments, we demonstrate that (i) SOff-AMS substantially increases the extractable fraction of organic carbon that is quantitatively analyzed constraining both primary (e.g., fossil-fuel combustion) and secondary (e.g., oxygenated organic aerosol) sources; (ii) the SOff-AMS-based OA spectra compare well with online measurements suggesting that online-level characterization can be achieved offline with this technique, and; (iii) water-insoluble coarse OA fraction of ambient PM can also be quantitatively characterized via this technique, which has traditionally been challenging to study via both online and offline methods. Overall, SOff-AMS is a powerful offline technique to comprehensively characterize ambient OA. It can considerably expand the reach of atmospheric chemistry studies to diverse urban and rural locations worldwide, where online measurements with advanced mass spectrometers are usually not feasible.

## 2 Materials and Methods

### 2.1 Sample Collection

A total of 8 multiseason filter samples were collected for these tests in Magadino (Switzerland) and Krakow (Poland). In Magadino, PM_2.5_ and PM_10_ samples were collected on 1 January 2019 and 27 June 2018 at the Magadino-Cadenazzo site (part of the Swiss National Air Pollution Monitoring Network – NABEL), while in Krakow, PM_1_ and PM_10_ samples were collected on 26 January 2018 and 27 June 2018 on the roof of the Physics and Applied Computer Science Faculty of the AGH University building (20 m.a.g.). Some additional 24 h fine PM filters were also collected on other days for different purposes; here, only the water-soluble organic carbon and total organic carbon analyses are used (performed with the same protocols as for samples in this study, details below). The sampling occurred on quartz filters using high volume samplers at a nominal flow rate of 0.5 m^3^ min^–1^ for 24 h each (00–23.59 h).

### 2.2 SOff-AMS Analysis

SOff-AMS builds on prior Off-AMS work by replacing Milli-Q water with organic solvents for sample extraction.^20,21^ High-purity acetone (Sigma-Aldrich, CAS:67–64–1; ≥99.8% purity) and ultrahigh-purity methanol (Sigma-Aldrich, CAS:67–56–1; ≥99.92% purity) were used for this purpose. These solvents were selected since their use for preparing filter sample extracts is well-documented in previous studies.^17,23,30–35^ Filter samples extracted in ultrapure Milli-Q water were also measured for reference. In total, 24 sample extracts were prepared including both sites -8 in acetone, 8 in methanol, and 8 in water. The Krakow winter samples were extracted using 4 punches of 16 mm diameter per extract, while the remaining samples were extracted using 5 punches per extract. The punches were dissolved in 20 mL of organic solvent or Milli-Q water in a glass vial by sonicating for 20 min at 30 °C, followed by vortex treatment for 1 min. Subsequently, the vortexed extracts in organic solvents were filtered using PTFE syringe filters (Infochroma; product # 8813Y-*P*4̅; pore size: 0.45 μm) attached to a glass syringe (SOCOREX Dosys # 155.0310). The glass syringes and PTFE filters were replaced with PTFE syringes and nylon filters (Infochroma; product # 8813Y–N-4; pore size: 0.45 μm) for water extracts to be consistent with the prior water-based extraction procedure.^21^ All filter extracts were spiked with 250 μL of 200 ppm of isotopically labeled ammonium nitrate and ammonium sulfate solution (^15^NH_4_^15^NO_3_, (^15^NH_4_)_2_^34^SO_4_) used as internal standard. The glass vials and glass syringes were extensively rinsed prior to use with Milli-Q water, followed by HPLC-grade acetone and methanol, and heated at 100 °C in a furnace for 14 h to eliminate background contamination. The PTFE syringes were rinsed with 120 mL of Milli-Q water prior to use but were not heat-treated. The PTFE filters were rinsed with ∼100 mL of sample extraction solvent. Filter blanks and pure solvents spiked with the internal standard were also measured following the same protocols.

The extracts were nebulized using synthetic air via a microflow nebulizer (Apex 2.0; Elemental Scientific Inc.) at a flow rate of 0.7 slpm into a cyclonic spray chamber. The chamber was maintained at 60 °C for water extracts and at 25 °C for methanol and acetone extracts to achieve a stable AMS signal during measurements. The excess solvent was condensed out by a Peltier-cooled condenser maintained at 2 °C and evacuated using an inbuilt peristaltic pump in the nebulizer. For water extracts, a Nafion dryer (Perma Pure LLC) was additionally installed downstream of the nebulizer. The sample spray was diluted with 2.1 slpm, 2.8 slpm, and 5 slpm of clean airflow for water, methanol, and acetone extracts, respectively. The dilution ratios were established following several trials to optimize the stability of the AMS signal during sample measurements. The resulting aerosols were measured via a high-resolution long-time-of-flight aerosol mass spectrometer (LToF-AMS) (Aerodyne Research Inc.). The operating principles of the AMS instrument are described in detail by DeCarlo et al.,^36^ and the offline protocols are thoroughly discussed elsewhere.^21,37,38^

Two sets of stainless-steel transfer lines were used between the nebulizer and the AMS, one set reserved for organic solvents and the other for water extracts. These steps were taken to avoid cross-contamination between solvents- and water-based measurements and to maintain a clean background. Samples extracted in different solvents were analyzed in separate batches. The transfer lines were flushed thoroughly for several hours before switching between the batches. The analysis sequence began by measuring spiked solvent, followed by extracts (Figure 1, filter blanks were measured following the same sequence). During analysis, a 6-to-8 min solvent flush was performed between samples to eliminate potential memory effects and ensure a low background signal between sample runs. Depending on the solvent, OA of the least polluted sample measured by the AMS in this study (i.e., summertime Magadino PM_2.5_) exceeded spiked solvent blank by a factor of 5–12 and by 21–25 for the more polluted winter samples (Figure 1). The AMS data were analyzed using Squirrel (v1.64) and Pika (v1.24) modules on the IGOR Pro software platform (Wavemetrics, Inc., Portland, OR). The high-resolution ion fragments were fitted to peaks between *m*/*z* 12 and 150 in each mass spectrum. The signals beyond *m*/*z* 150 were analyzed at unit mass resolution (UMR). Finally, the two sections of the mass spectra were combined to obtain a complete organic mass spectrum. For the samples from Krakow, the OA spectra from SOff-AMS were also compared with data collected from a colocated online Q-ACSM (PM_1_ inlet, standard vaporizer, Aerodyne Research Inc.), averaged over a 24 h period corresponding to the filter collection date.^39^ This allowed us to assess the effectiveness of SOff-AMS in replicating real-time characterization of ambient OA. In addition, the least and most polluted samples used in this study (i.e., summertime Magadino PM_2.5_ and wintertime Krakow PM_10_, respectively) were stored in the dark at 4 °C and remeasured after 36 h to check for stability in aerosol spectra.

**Figure 1:**
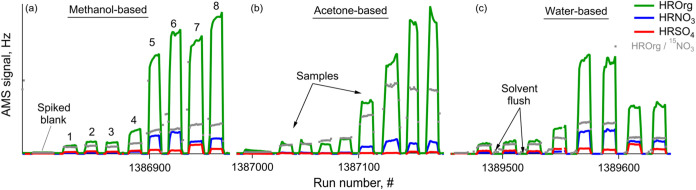
Example timeseries of HROrg, HRNO_3_, and HRSO_4_ of filter samples extracted in (a) methanol, (b) acetone, and (c) water during SOff-AMS measurements. The labels 1–4 indicate summer ambient samples including Magadino: (1) PM_2.5_ and (3) PM_10_, and Krakow: (2) PM_1_ and (4) PM_10_. Winter samples included Magadino: (5) PM_2.5_ and (6) PM_10_, and Krakow: (7) PM_1_ and (8) PM_10_. The sampling order (1–8) in (a) methanol tests is replicated in (b) acetone and (c) water measurements.

### 2.3 AMS Data Treatment

The combined organic AMS spectrum (HR *m*/*z* 12–150 and UMR *m*/*z* 151–467) extracted from samples was directly quantified from AMS measurements using eq 1, which is discussed in detail by Casotto et al.^27^

1 $${\mathrm{OA}}_{\mathrm{AMS},i,m/z}\left(\mu gm^{-3}\right)=\frac{I_{\mathrm{AMS},i,m/z}}{{{\mathrm{RIE}}_{\mathrm{org}}}^{*}c}.\frac{1}{I_{{i,}^{15}NO_{3}}}\cdot M_{\mathrm{IS}}\cdot \frac{A}{A_{p}}\cdot \frac{1}{V_{a}}$$

Here, OA_ams,i,*m*/*z*_ is the organic aerosol concentration in μg m^–3^ of an ion in question (*m*/*z*) for a sample or blank “i”. *I*_AMS,*m*/*z*_ is the average signal from the AMS of an ion (*m*/*z* ≤ 150: HR, *m*/*z* > 150 UMR) in units of ion frequency, *I*_i,^15^NO_3__ is the labeled nitrate signal measured as the sum of the frequencies of ^15^NO^+^ and ^15^NO_2_^+^, and M_IS_ is the mass of labeled nitrate added as internal standard to each sample calculated as shown in eq 2.

2 $$M_{\mathrm{IS}}=M_{\mathrm{IS}}^{\prime }\cdot \frac{M_{\mathrm{IS}}\left(j15NO_{3}\right)}{M_{\mathrm{IS}}\left(NH_{4}j15NO_{3}\right)}$$

Here, *M*_IS_^’^ is the total mass of internal standard injected, and M_IS_(^15^NO_3_)/M_IS_(NH_4_^15^NO_3_) is the mass fraction of labeled nitrate in the internal standard. *A* is the total filter area used in sample collection, *A*_p_ is the filter area punched for sample extraction, and *V*_a_ is the total volume of air sampled during filter collection. For all filter samples, ion concentrations calculated via eq 1 were filter background-corrected prior to further analysis using a pure solvent measurement that was spiked with an internal standard.

Returning to eq 1, RIE_org_ is the default literature relative ionization efficiency of organics in the AMS taken 1.4. However, the AMS used in the present study operated with an usually high RIE for organics, represented here as the product of RIE_org_ and the correction factor “*c*” (this formulation is chosen to emphasize that the organic RIE estimated herein represents the unusual performance of the specific instrument used and should not be held generally true for all AMS instruments). To estimate *c*, we assumed that the RIE of organics is equal to the RIE of levoglucosan. Previous studies have shown the RIE of levoglucosan to be 1.4–1.8 for AMS and 1.27–1.4 for ACSM, which is consistent with the typical organics RIE of 1.4.^40,41^ This allowed us to calibrate for deviations in our AMS instrument relative to others. We determined c to be 1.86 ± 0.1, as shown later in Figure 2a.

**Figure 2:**
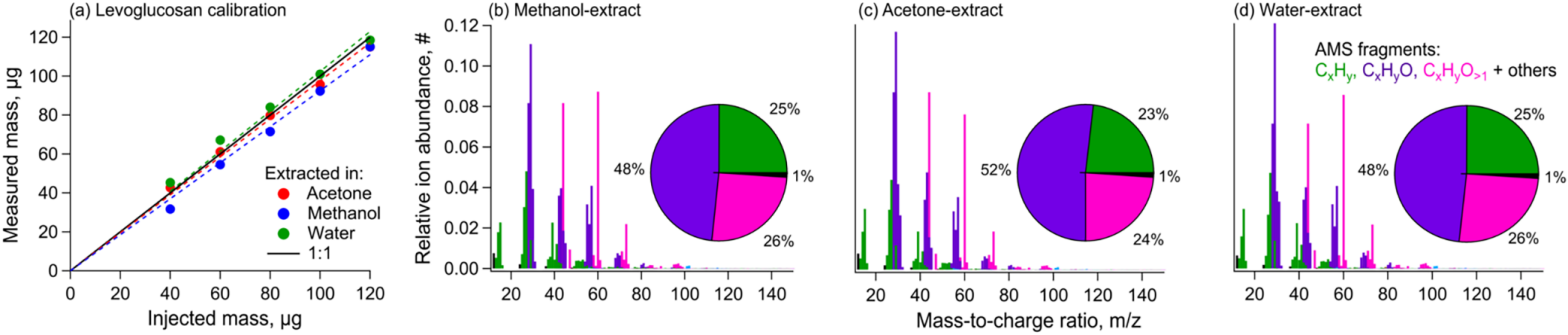
(a) Calibration curves for levoglucosan prepared in acetone, methanol, and water. (b–d) AMS spectra of levoglucosan OA aerosolized in (b) methanol, (c) acetone, and (d) water. Note: Panel (a) shows slope-adjusted linearity in instrument response to levoglucosan with a calculated average correction factor of 1.86.

The measurement errors after background correction were propagated based on eq 3 using the standard deviation in spectra recorded over the measurement period of a filter sample (σ_OA,i,*m*/*z*_) and spiked solvent blank (σ_OA,B,*m*/*z*_), processed with eq 1

3 $${\sigma \mathrm{OA}}_{\mathrm{AMS},i,m/z}\left(\mu gm^{-3}\right)=\sqrt{{\left(\sigma_{OA_{\mathrm{AMS},i,m/z}}\right)}^{2}+{\left(\sigma_{OA_{\mathrm{AMS},B,m/z}}\right)}^{2}}$$

The OA concentration was calculated as the sum of all organic aerosol fragments

4 $${\mathrm{OA}}_{\mathrm{AMS},i}\left(\mu gm^{-3}\right)=\sum_{m/z}OA_{\mathrm{AMS},i,m/z}$$

To compare to reference measurements, we further calculated the OC concentration as the sum of all organic aerosol ions divided by the bulk organic matter-to-organic carbon ratio (OM/OC). OM/OC was calculated using the improved-ambient method based on Canagaratna et al. implemented in the analytic procedure for elemental separation *(*APES) v1.09 module built on the IGOR Pro software platform.^42^

5 $${\mathrm{OC}}_{\mathrm{AMS},i}\left(\mu gm^{-3}\right)=\frac{\sum_{m/z}OA_{\mathrm{AMS},i,m/z}}{{\left(\mathrm{OM}/\mathrm{OC}\right)}_{i}}$$

We proceeded analogously for inorganic constituents. Nitrate was quantified using the total AMS signal of ^14^NO^+^ and ^14^NO_2_^+^ ions instead of *I*_AMS,*m*/*z*_, and similarly ^32^SO^+^ and ^32^SO_2_^+^ and their isotopically labeled counterparts (^34^SO^+^ and ^34^SO_2_^+^) were used for sulfate. Since the RIE for the pairs ^14^NO_3_ – ^15^NO_3_ and ^32^SO_4_ – ^34^SO_4_ can be assumed to be the same, the RIE term was omitted from the equation.

### 2.4 Reference Analyses of Water-Soluble and Total Organic Carbon

The WSOC content in the filter samples was measured with a total organic carbon (TOC) analyzer (TOC-L_CPH_; Shimadzu), extracted analogously to those for AMS analyses. The organic carbon constituents were oxidized to CO_2_ using hydrochloric acid and measured using a “680 °C combustion catalytic oxidation with nondispersive infrared (NDIR) detection” technique. Given the nature of the solvent, no analogous analyses could be performed for methanol and acetone extracts. In addition, the total organic carbon (OC_total_) and elemental carbon (EC) content of filter samples was measured via the thermo-optical transmittance (TOT) method, using a Lab OC-EC Aerosol Analyzer (Model 5 L, Sunset Laboratory Inc.) and adhering to the EUSAAR-2 protocol. Quartz filter samples undergo heating up to 650 °C in a helium-rich atmosphere initially, followed by further heating up to 850 °C in a mixture of 2% oxygen gas in helium, using the controlled heating ramps specified in the EUSAAR-2 thermal protocol.^43^ During this process, OC evolves in the inert atmosphere, while EC undergoes oxidation in the helium–oxygen atmosphere. Charring correction is implemented by monitoring the sample transmittance throughout the heating process. The limit of detection (LOD) for TOT analysis was 0.02 μg m^–3^ of carbon. The field blanks were prepared and processed following identical procedures. For optimal comparability between the samples, all samples were measured on the same day in the same laboratory. The uncertainty is calculated at 15% for OC and 23% for EC and takes into account the limits of detection, reproducibility, repeatability, and precision based on references, the detector sensitivity uncertainty, and the area uncertainty of the filter.

## 3 Results and Discussion

### 3.1 Calibration and Limits of Detection

Five-point calibrations were performed for all solvents using mixes prepared with levoglucosan and inorganic standards (ammonium nitrate and ammonium sulfate) (Figures 2 and S1). The mixes were spiked with the internal standard, nebulized, and measured using the same method as for sample extracts. The calibration slopes showed 80–90% recovery of the injected concentrations for inorganic standards. The slope of the levoglucosan calibration was used to determine the corrected organic RIE (RIE_org_ * *c*) as discussed previously (this correction is already applied in Figure 2a, yielding data in good agreement with the 1:1 line). With the corrected RIE applied,^36^ OC_ams_ compared well with the reference OC measured using the TOC analyzer.

The solvent background-subtracted levoglucosan spectra were strongly similar across solvents (cosine angles: 9–13°) with strong signals at characteristic *m*/*z* 60 and 73 contributed by C_2_H_4_O_2_^+^ and C_3_H_5_O_2_^+^ fragments (Figure 2).^44,45^ Ion intensities of small molecular weight fragments (<*m*/*z* 80) including C_*x*_H_*y*_ and other oxygenated ions that formed majority of the total spectra were also comparable between all solvents (Figure S2). Some differences were observed beyond *m*/*z* > 80; larger C_*x*_H_*y*_ fragments were more prominent in methanol and acetone extracts relative to water, while oxygenated ions showed higher signal in water relative to acetone and were comparable in methanol. These ions constituted less than 1% of the total spectra. Artifacts introduced by imperfect solvent background subtractions were evaluated by comparing the resulting levoglucosan mass spectra to a best-estimate spectrum. The best-estimate spectrum was calculated as the average spectrum of the three solvents for the highest levoglucosan concentration (6 μg mL^–1^). For each fragment ion, data from solvents exhibiting background subtraction imperfections, such as residual solvent signals at low levoglucosan concentrations, were excluded. Thereby, we found that mainly fragment ions that are major contributors to the solvent background were affected in the levoglucosan mass spectrum (Figures S3 and S4), with specific influences from acetone (C_2_H_3_O^+^- and CO_2_-related peaks), methanol (CH_3_O^+^), and water (CO_2_^+^). However, these effects had only a minor impact on the OM/OC ratio (within 4% of the best-estimate value: Figure S5) and the determined mass concentration (within 10% of the true concentration: Figure S6). Furthermore, we also investigated solvent removal from four mixtures containing different concentrations of inorganic standards (2–5 μg mL^–1^ NH_4_^14^NO_3_; 2.5 μg mL^–1^ NH_4_^15^NO_3_; 2–5 μg mL^–1^ NH_4_^32^SO_4_; 2.5 μg mL^–1^ NH_4_^34^SO_4_) that showed no considerable change in organic aerosol detected by the AMS (∑_*m*/*z*_ *I*_AMS,i,*m*/*z*_) with increasing salt concentrations (Figure S7).

The levels of OC_ams_ for spiked solvent procedural blanks were 1.4 μg mL^–1^ (29 μg) in acetone, 0.6 μg mL^–1^ (11 μg) in methanol, and 0.2 μg mL^–1^ (4.9 μg) in water. Based on these analyses, we estimate the procedural limits of detection (LoD_SB_, defined here as 3 standard deviations of the spiked solvent procedural blank) of OC_ams_ were 0.8 μg mL^–1^ (16 μg) in acetone, 0.15 μg mL^–1^ (3 μg) in methanol, and 0.2 μg mL^–1^ (4 μg) in water. Without solvent background subtraction, the measured OC_ams_ of filter blanks (FB) were comparable for methanol (0.6 μg mL^–1^:11 μg) and 1.7 times higher for acetone (2.5 μg mL^–1^: 50 μg) and 1.5 times higher for water (0.3 μg mL^–1^: 7 μg). Thus, the procedural blanks dominated the OC_ams_ measured for the filter blanks. The LoD_FB_ of OC_ams_ was defined here as 3 standard deviations of the filter blank analyses, i.e., it describes the minimum amount of OC_ams_ required from an atmospheric aerosol filter sample. LoD_FB_ was 1.3 μg mL^–1^ (26 μg) in acetone, 0.03 μg mL^–1^ (0.6 μg) in methanol, and 0.05 μg mL^–1^ (1 μg) in water. These were substantially lower than the sample with the lowest solvent background-subtracted OC_ams_ (PM_2.5_ Magadino summer) with 3.9 μg mL^–1^ (78 μg) in acetone, 4.2 μg mL^–1^ (83 μg) in methanol, and 3.4 μg mL^–1^ (68 μg) in water.

Each solvent showed a distinct mass spectrum dominated by its own fragments. While C_2_H_3_O^+^ and C_3_H_6_O^+^ were prominent in acetone spectra alongside some C_*x*_H_*y*_ fragments, methanol spectra showed strong prevalence of CH_3_O^+^, CH_4_O^+^, and CHO^+^ ions (Figure S8). Several C_*x*_H_*y*_ fragments were commonly observed in acetone, methanol, and water indicating contamination artifacts, though minor. The spectra of filter blank extracts prior to solvent background subtraction were also very similar to spiked solvents (Figure S9). These factors further support that the filter material had a minor influence on the measurement background, which mainly originated from impurities in solvents or the experimental setup. Henceforth, all analyses are filter blank corrected unless stated otherwise. Methanol is frequently used for sample extractions in other analytical techniques and is shown to extract diverse chemical species given a midrange polarity index of 0.7.^17,46,47^ It also has considerably lower vapor pressure than acetone (boiling points: 64.7 °C methanol vs 56 °C acetone) that prevents samples from uncontrolled concentrating via solvent evaporation prior to being nebulized into the spray chamber. For these reasons, we focus the discussion on methanol while providing analogous comparisons for acetone- and water-based measurements.

### 3.2 Bulk OC Solubilities of Ambient OA

The bulk OC solubility was measured directly from the AMS for all solvent extracts as OC_ams_ using eq 5 and also via the nonpurgeable organic carbon (NPOC) analysis of water extracts. All summer and Magadino winter water extracts showed high NPOC content, while Krakow winter samples were less soluble (Figure 3). Overall, the water solubilities of samples analyzed in this study were largely representative of other days in the same season for each site (Figures 3 and S10). Furthermore, while the OC solubility in wintertime Krakow was lower than in other regions in Europe, mainly due to high coal combustion for residential heating, the solubilities of summertime Krakow and all Magadino samples were comparable to other European sites.^48–50^ The contributions of dominant OA sources in Krakow and Magadino, i.e., HOA, solid-fuel combustion-related primary OA, and oxygenated OA, were also similar to other European locations (Figure S11). Thus, the samples investigated in this study represented typical OA source profiles and bulk OA water solubility across Europe and likely also to other regions, e.g., Asia.^20,51,52^

**Figure 3:**
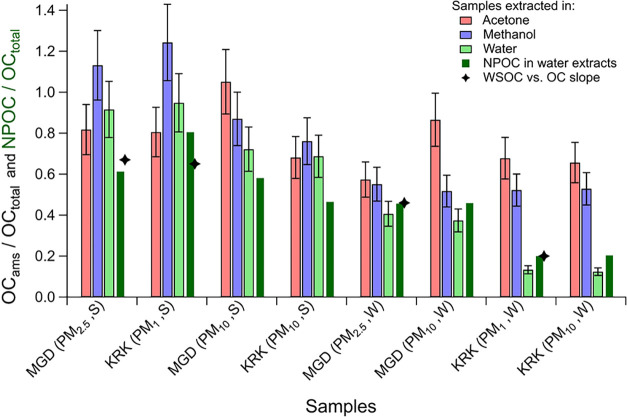
Fractions of organic carbon extracted from filter samples in acetone (red), methanol (blue), and water (light green) extracts measured via SOff-AMS. The NPOC fraction in water extracts is also shown (dark green). Black star markers indicate season-based slopes of WSOC vs OC distributions for all fine PM samples collected in Krakow and Magadino (Figure S10). On the *x*-axis, the samples are labeled denoting sites, particle sizes, and seasons in which the samples were collected. “MGD” (Magadino) and “KRK” (Krakow) are sampling locations. PM_10_, PM_2.5_, and PM_1_ are particle sizes, and “S” and “W” indicate summer and winter seasons.

OC_ams_ for water extracts showed a comparable trend to that of NPOC, but all summer samples exceeded corresponding NPOC values. The uncertainties in organic mass-to-organic carbon ratios (OM/OC) and field blank subtractions at low summer concentrations or loss of purgeable inorganic carbon (e.g., carbonates) during acid dissolution of sample extracts in NPOC analyses could explain the higher OC_ams_. In general, OC was more soluble in organic solvents compared to water (OC_ams,os_/OC_ams,w_) (Figure 3). OC_ams,os_/OC_ams,w_ ranged 1.1–4.2 for methanol and 1–5.2 for acetone extracts. For both sites, the ratio strongly varied between seasons and particle sizes. For fine OA, winter OC_ams,os_/OC_ams,w_ (2.9 ± 1.6) was a factor of 2.5 higher than summer (1.2 ± 0.2). For PM_10_ OA, the ratio increased to 3.3 ± 1.5 for winter and 1.3 ± 0.2 for summer samples. The wintertime OC dissolved more in organic solvents, likely due to increased contributions from hydrocarbon-like or other fresh aerosols. This is further evidenced by a high fraction of elemental carbon in winter samples (16–20%), suggesting major contributions from combustion-based nonwater-soluble emissions (Figure S12). Coal combustion POA is an important source in Krakow during winter that contributed 12% to fine OA, alongside 3% from biomass burning POA.^27^ The OC solubility of Krakow samples enhanced by a factor of 2.8–3.2 in organic solvents relative to water, suggesting substantially increased dissolution of coal combustion-related OA. Coal combustion did not contribute significantly to POA in Magadino, although biomass burning is an important source.^19,53^

The OC solubilities also varied between methanol and acetone extracts. For all winter samples, 53–57% of OC_total_ was soluble in methanol exceeding 14–40% in water. The solubility increased further in acetone (67–91%) compared to methanol due to increased dissolution of nonpolar OA constituents. The relatively consistent solubility of winter samples in methanol could be attributed to its midscale polarity, permitting the extraction of diverse chemical species. Acetone and water have more separated polarities potentially causing dissolution of varying fractions of organic mass strongly depending on OA composition. In comparison, summer samples showed high OC solubilities in all solvents including (70–95%) water, (80–120%) methanol, and (80–130%) acetone due to greater oxygenation of constituting organic compounds. Overall, the bulk OC measurements demonstrated that SOff-AMS can significantly enhance the OC mass fraction that can be analyzed offline from filters, especially during wintertime conditions.

### 3.3 Solubility of OA Constituents in Organic Solvents Versus Water

The OA spectra differed between sites and seasons depending on influencing factors including differences in precursor emissions, environmental conditions, ambient OA levels, and their solubility in the extraction solvent. The spectra of OA extracted in organic solvents captured these differences more effectively than water extracts since several mass fragment groups were relatively enhanced. The methanol and acetone extracts of winter OA were dominated by C_*x*_H_*y*_ fragments that constituted over 50% of the HR spectra with smaller contributions from oxygenated species (36–40%) (Figures S13 and 14). In contrast, only ∼30% of each spectrum from water extracts was constituted by C_*x*_H_*y*_ fragments. For each winter sample, extracts in organic solvents produced largely comparable spectra that were dissimilar to water extracts (cosine: 35–50°). In the UMR region spanning *m*/*z* 151–467, wintertime OA spectra were substantially enhanced in organic solvents (Figures 4 and S15). The UMR signal constituted 25–28% (methanol) and 27–30% (acetone) of the winter OA spectra in Krakow, which reduced to 6–7% in water extracts. For Magadino winter, this fraction reduced to 11% in both methanol and acetone and was 7% in water extracts (Figure S13). The Magadino HR spectra were comparable across solvents. However, methanol extracts were slightly more similar to water than acetone (<15 vs <20°). Overall, these observations indicated lower water-insoluble primary source contributions (coal combustion and traffic exhaust) in Magadino than in Krakow during winter.

**Figure 4:**
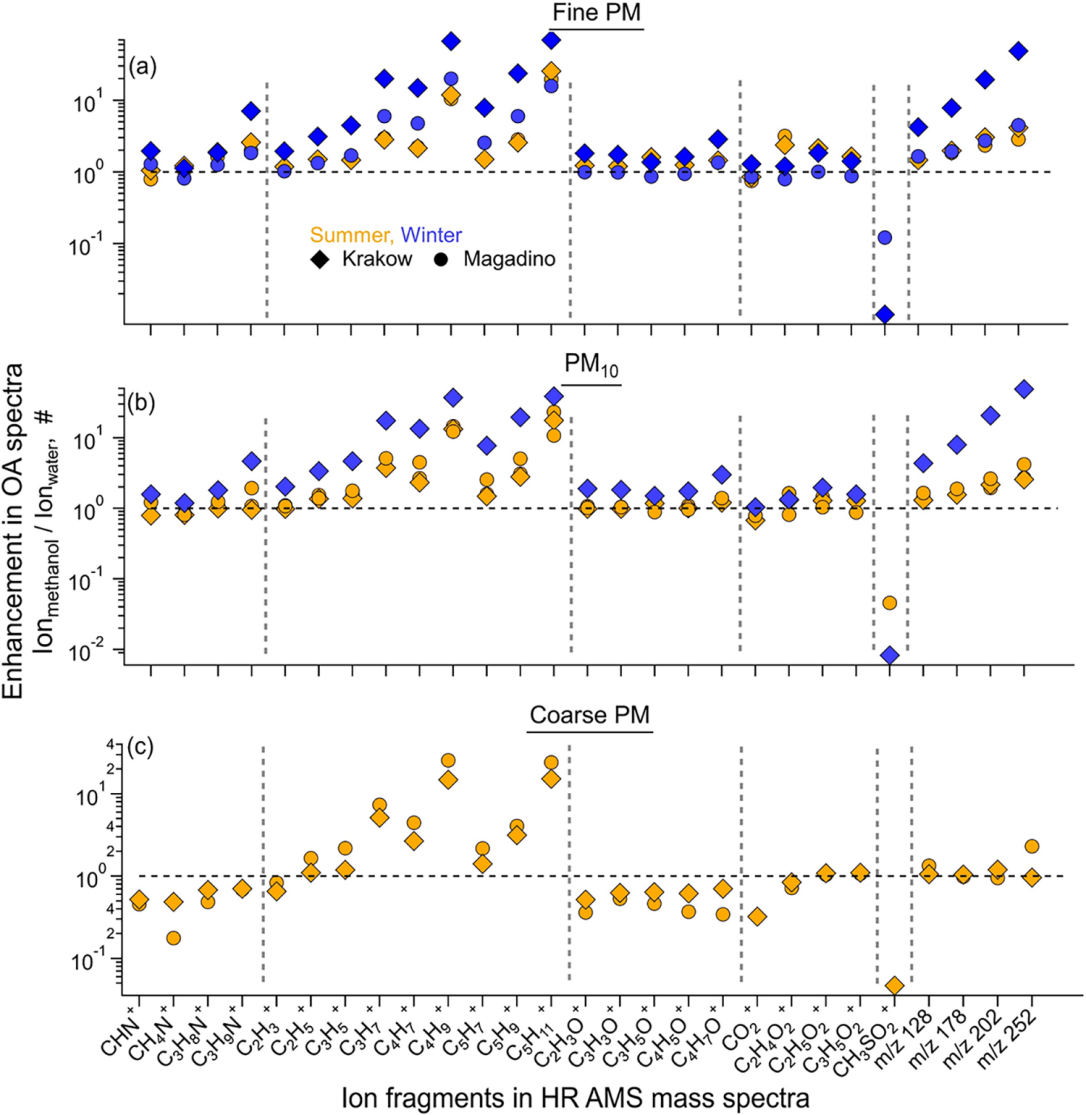
Enhanced contributions in methanol-SOff-AMS measurements relative to water-Off-AMS of key ions to (a) fine (PM_1_ and PM_2.5_), (b) PM_10_, and (c) coarse PM spectra from summertime (yellow) and wintertime (blue) Krakow and Magadino.

More broadly, over all sites and seasons, oxygenated fragments constituted 60 ± 2% of the HR spectra from water extracts, and their OM/OC ratio also remained stable (2.03 ± 0.05). Acetone extracts attributed 48 ± 5% to C_*x*_H_*y*_ fragments, and the OM/OC was considerably lower across seasons and sites (1.6 ± 0.09) (Figure S16). Thus, acetone- and water-based sample extractions indicated a certain solubility bias that would benefit a more targeted analysis of select OA constituents. On the other hand, methanol extracts captured variations in OA composition over a broader range of C_*x*_H_*y*_ (37–54%), oxygenated fragments (41 – 58%), and OM/OC (1.8 ± 0.14).

Several key ions were also enhanced in organic solvents (Figures 4 and S17–S19). The small hydrocarbon fragments including C_2_H_3_^+^, C_2_H_5_^+^, C_3_H_5_^+^, C_3_H_7_^+^, C_4_H_7_^+^, C_4_H_9_^+^, C_5_H_7_^+^, C_5_H_9_^+^, and C_5_H_11_^+^ increased by a factor of 1.5–60 in spectra from both Krakow and Magadino. The oxygenated fragments (e.g., C_2_H_4_O_2_^+^, C_2_H_5_O_2_^+^, among others) were comparatively less yet clearly enhanced (1.2–2.7). Within each category of fragment species (e.g., C_*x*_H_*y*_, C_*x*_H_*y*_O_*z*_), higher molecular weight ions showed greater enhancement than smaller ones, likely due to higher hydrophobicity and increased solubility of larger-molecular-weight species from hydrocarbon-like OA. However, reduced nitrogen-containing fragments exhibited a mixed behavior. C_3_H_8_N^+^ and C_3_H_9_N^+^ that are known tracers of cigarette smoke increased considerably (1.5–6), while CHN^+^ and CH_4_N^+^ were comparable in methanol and water but reduced in acetone extracts. Similarly, sulfur-containing fragments −CHSO^+^ and CH_3_SO_2_^+^ were reduced by 1–2 orders of magnitude in organic solvents (Figures 4, S17 and S18). Consequently, it may be more difficult with SOff-AMS to apportion sources traced via these fragments (e.g., methanesulfonic acid; marine SOA), yet possible with clean backgrounds and application of other potential markers that might be enhanced.

The signal enhancement with molecular weight was also observed in the UMR region of the spectra, which was dominated by PAHs. The signal increased consistently from *m*/*z* 128 (4–5) to 252 (40–60) for winter OA in organic solvents. Acetone extracts showed slightly higher ion signal than methanol in this mass range due to lower polarity.^39,40^ Summer samples also showed a similar trend for all fragment categories but with weaker enhancement relative to winter (Figures 4 and S17), likely due to reduced contributions from PAHs.

The magnitude and composition of coarse PM were calculated as the difference between the spectra of PM_10_ and fine PM. While OC_coarse_ was within the measurement error of OC_fine_ for the winter samples indicating minimal to no coarse contributions, significant coarse OC was observed during summer (Krakow: 1.5 μg/m^3^, Magadino: 0.3 μg/m^3^ in methanol extracts). Thus, we focus the discussion on summertime coarse OA (Figures 4c and S17). At both locations, mainly select hydrocarbon fragments were considerably enhanced in methanol and acetone extracts relative to water, with a stronger increase for saturated (C_4_H_9_^+^, C_5_H_11_^+^, C_3_H_7_^+^) than for unsaturated fragments (C_4_H_7_^+^, C_5_H_9_^+^). The enhancement in acetone was a factor of 3–5 higher than in methanol. Consequently, observations over a larger sample size can likely differentiate sources of coarse PM between different measurement locations.

The limitations of using water as an extraction solvent are clear for winter OA, which produced very similar spectra for Krakow and Magadino despite differences in sources between the sites (Figures S13d, S20, and S21). Water extracts summertime OA more effectively than winter, which is also evidenced by their increased OC solubility (Figure 3). For these samples, the HR spectra (i.e., m/*z* ≤ 150) constituted >95% of the OA mass across all solvent extracts, 50–60% of which were contributed by oxygenated fragments (C_*x*_H_*y*_O and C_*x*_H_*y*_O_>1_).

We also compared our offline analyses of Krakow fine OA with measurements from a colocated online Q-ACSM that can be considered real-time characterization for our purposes in this study.^39^ The SOff-AMS spectra were integrated to unit mass resolution and renormalized after excluding large ions (*m*/*z* 126–467) since the Q-ACSM measured only up to *m*/*z* 125. This comparison should be treated with caution due to differences between AMS and Q-ACSM instruments. First, the longer sample/background switching interval of Q-ACSM instruments increases the importance of slow vaporization processes in the mass spectrum, which can enhance the contributions of ions such as *m*/*z* 44.^54^ Comparison of *m*/*z* > ∼100 should also be treated with caution due to the uncertainty in estimated *m*/*z* transmission function of the Q-ACSM, which may cause systematic under/overestimation of ions at high *m*/*z* relative to low *m*/*z*. Different sampling cutoff sizes (AMS: PM_2.5_ vs Q-ACSM: PM_1_) could also potentially affect the OA spectra, though minimal under polluted conditions.^55^ Therefore, the Q-ACSM spectrum should not be considered a “gold standard” comparison for SOff-AMS data; nonetheless, comparison remains instructive.

The methanol extract and Q-ACSM spectra were highly comparable during winter (cosine: 16–17°) with similar fractions of total signal at *m*/*z* 44 (f_44_: 0.03 vs ACSM: 0.06), 43 (f_43_: 0.05 vs 0.08), and 60 (f_60_: 0.01 vs 0.01) (Figure 5a). The acetone and water extracts were slightly less comparable, though not entirely dissimilar (cosine: acetone 21°, water 23°) (Figures S22–S24).

**Figure 5:**
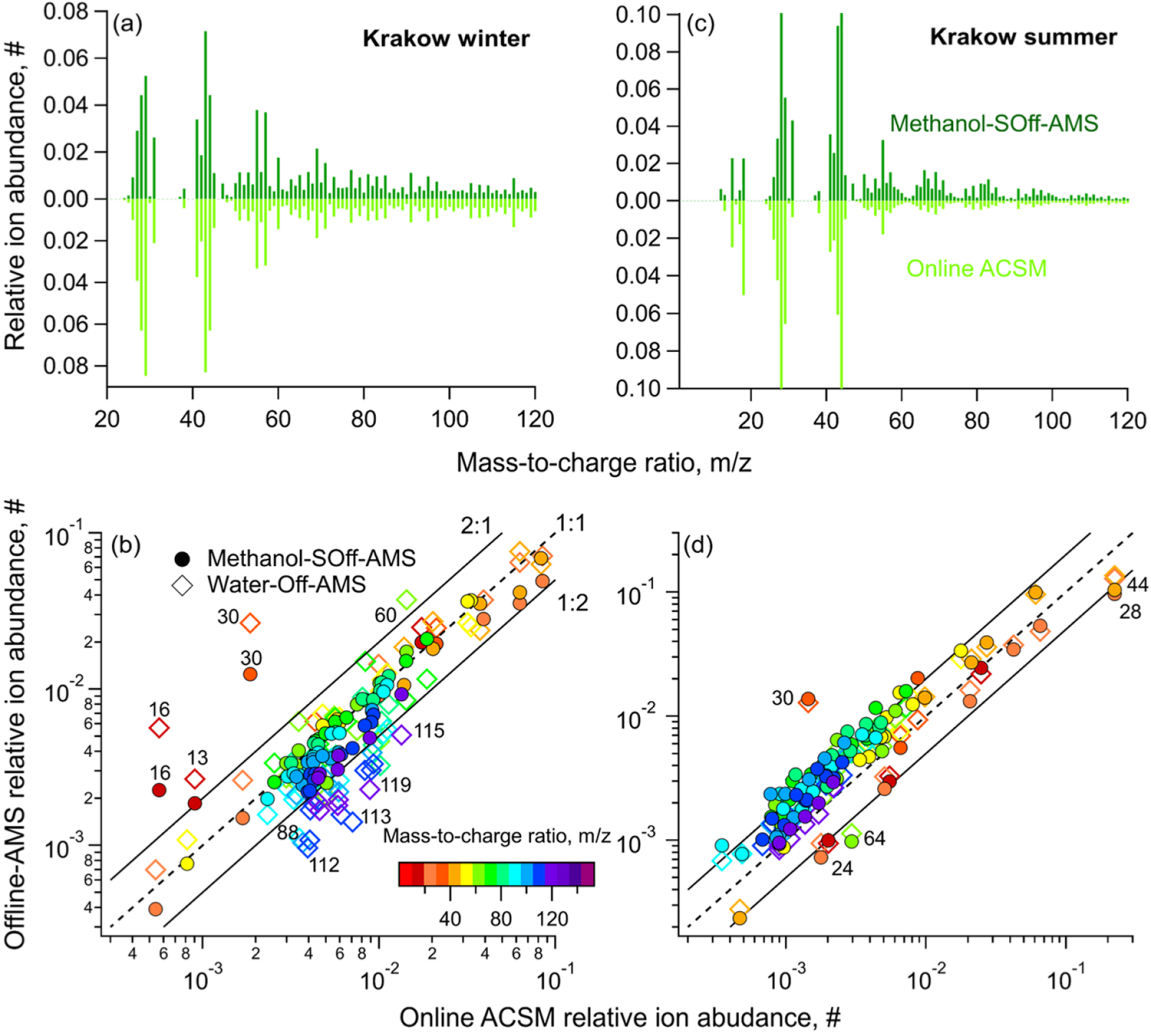
Comparisons of the (a) wintertime and (b) summertime Krakow PM_1_ OA spectra analyzed via SOff-AMS of methanol sample extract in the laboratory vs directly measured on site using a Q-ACSM. The OA spectra of Krakow PM_1_ samples measured in the laboratory from methanol and water-based sample extractions are compared for (c) winter- and (d) summertime conditions.

Summertime ACSM spectra were similar to the water extract (cosine: 18–22°), while methanol (28–30°) and acetone extracts (cosine > 40°) were considerably dissimilar (Figures 5d and S22 and 23). The similarity with water extract was strongly influenced by high signals at *m*/*z* 28 and 44 in ACSM spectra during summer. Upon excluding these ions (and the derived fragment ions 16, 17, 18), the ACSM spectra compared similarly to water (22–28°) as to methanol (22–24°) and acetone extracts (∼29°).

### 3.4 Implications for Ambient Measurements

We showed that SOff-AMS can characterize significantly larger mass fraction of wintertime OA with substantial primary- and less-aged source contributions in comparison to water extracts. Furthermore, the SOff-AMS spectra can replicate real-time measurements of ambient OA, demonstrating that characterization close to online-level can be achieved in offline laboratory-based measurements. Despite potential artifacts from sampling, incomplete solubility, and solvent interactions, the strong agreement between SOff-AMS and Q-ACSM measurements supports the reliability of SOff-AMS for OA characterization. The repeat analyses after 36 h also confirmed the stability of OA spectra in solvent extracts (Figure S25). Nevertheless, large-scale intercomparisons will be important to further evaluate its performance across diverse environments, sampling, and analysis periods. SOff-AMS enhanced the detection of several key markers of anthropogenic and biogenic sources in OA spectra, which can be coupled with source apportionment techniques to constrain sources of fresh ambient OA as well as other minor sources. For example, methanol extracts of Krakow winter OA not only showed markedly higher small hydrocarbon contributions relative to water extracts but also a substantially higher concentration of fragments typically related to polycyclic aromatic hydrocarbons (PAHs: 128, 152, 165, 178, 189, 202, 215, 226, 239, 252, and 276) (Figures 6 and S15).^56,57^ This is consistent with Krakow being strongly affected by fresh and only slightly aged residential coal combustion and wood burning emissions. Wood is the dominant solid fuel in wintertime Magadino, so the impact of hydrocarbons and PAHs was expectedly smaller (Figure 6) since wood burning emits less PAHs than coal combustion.^27,39^ This differentiation is crucial for constraining the relative importance of sources in Krakow, Magadino, and other areas that are impacted by fresh combustion and other nonwater-soluble emissions.^32,41^

**Figure 6:**
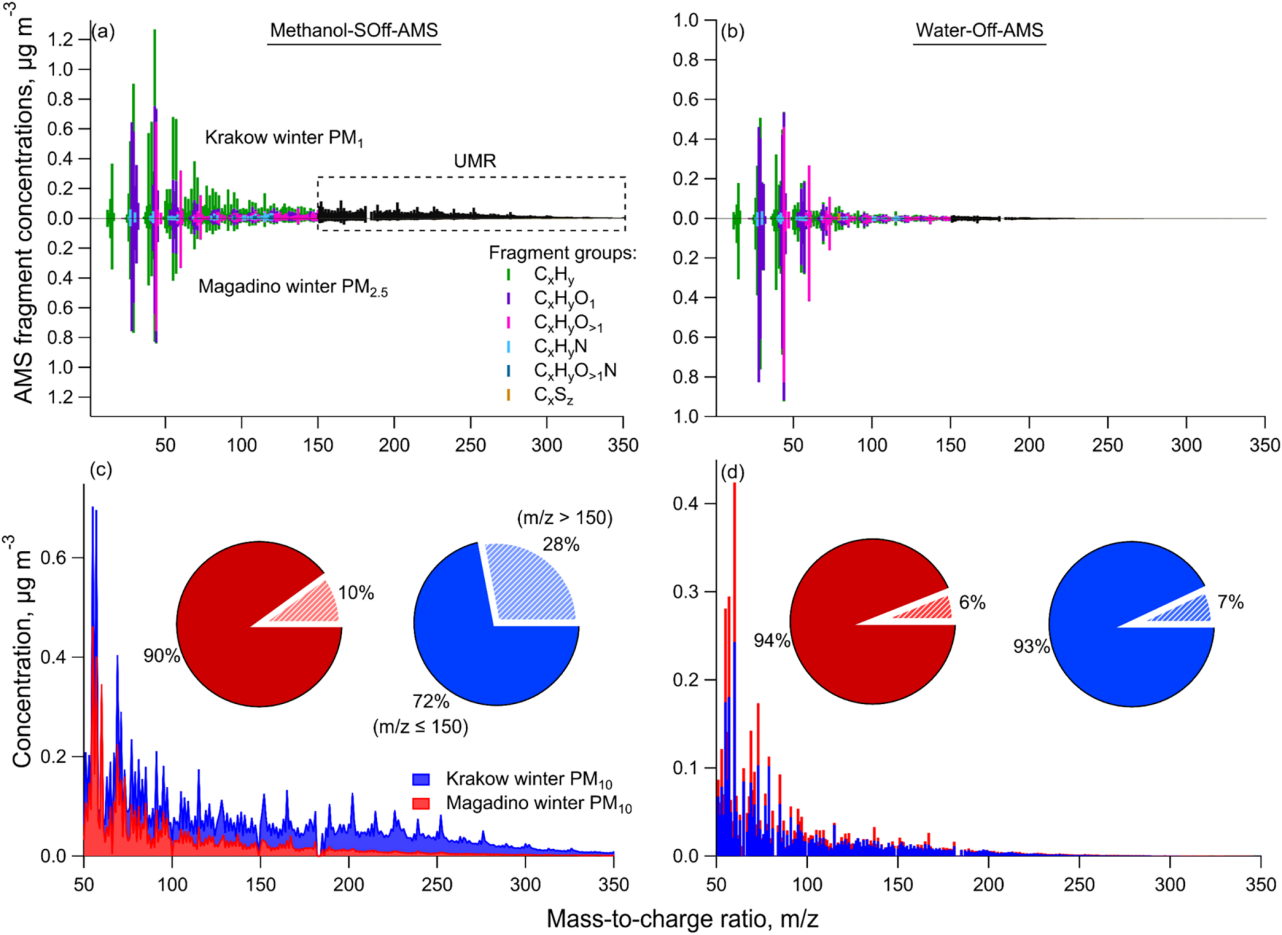
Mass spectra of wintertime fine OA from Krakow and Magadino measured via (a) methanol-SOff-AMS and (b) water-Off-AMS. Each spectrum combines high-resolution (≤*m*/*z* 150, colored) and UMR (>*m*/*z* 152, black) ion signals stacked on integer *m*/*z* values. (c, d) UMR spectra comparing the prevalence of higher molecular weight fragments in wintertime PM_10_ OA between Krakow and Magadino measured via (c) methanol-SOff-AMS and (d) water-Off-AMS. The pie charts (inset c, d) show the fraction of extracted aerosol mass of PM_10_ for m/*z* ≤ 150 and *m*/*z* > 150.

In the absence of a detectable amount of OC_coarse_ in winter, we focused on the chemical composition of summertime coarse OA (Figures 7 and S26). In general, acetone extracted a higher fraction (60–100%) than methanol and water extracts (35–49%) of the OC_coarse_ measured directly with the Sunset OC analyzer. Previous work based on water extracts has established that AMS fingerprints of primary biological organic aerosol such as pollen and fungal spores exhibit characteristic peaks C_2_H_5_O_2_^+^ and C_2_H_4_O_2_^+^ with approximately similar intensity in the aerosol spectra. We found this pattern in coarse OA from summertime Krakow and Magadino in both methanol and water extracts, however, with different C_2_H_5_O_2_^+^/C_2_H_4_O_2_^+^ ratios. Overall, the spectra showed clear differences between the extracts, as shown in Figure 7. Negative peaks at CH_2_O^+^, CH_3_O^+^, and CH_4_O^+^ for methanol extracts are the dominant peaks in solvent blanks and are removed due to their high uncertainty. The coarse OA spectra from water extracts were characterized by a higher prevalence of oxygenated fragments relative to methanol extracts that showed a stronger presence of C_*x*_H_*y*_ fragments. This suggests that SOff-AMS can characterize primary coarse OA to potentially constrain both natural and anthropogenic sources (e.g., plastics).

**Figure 7:**
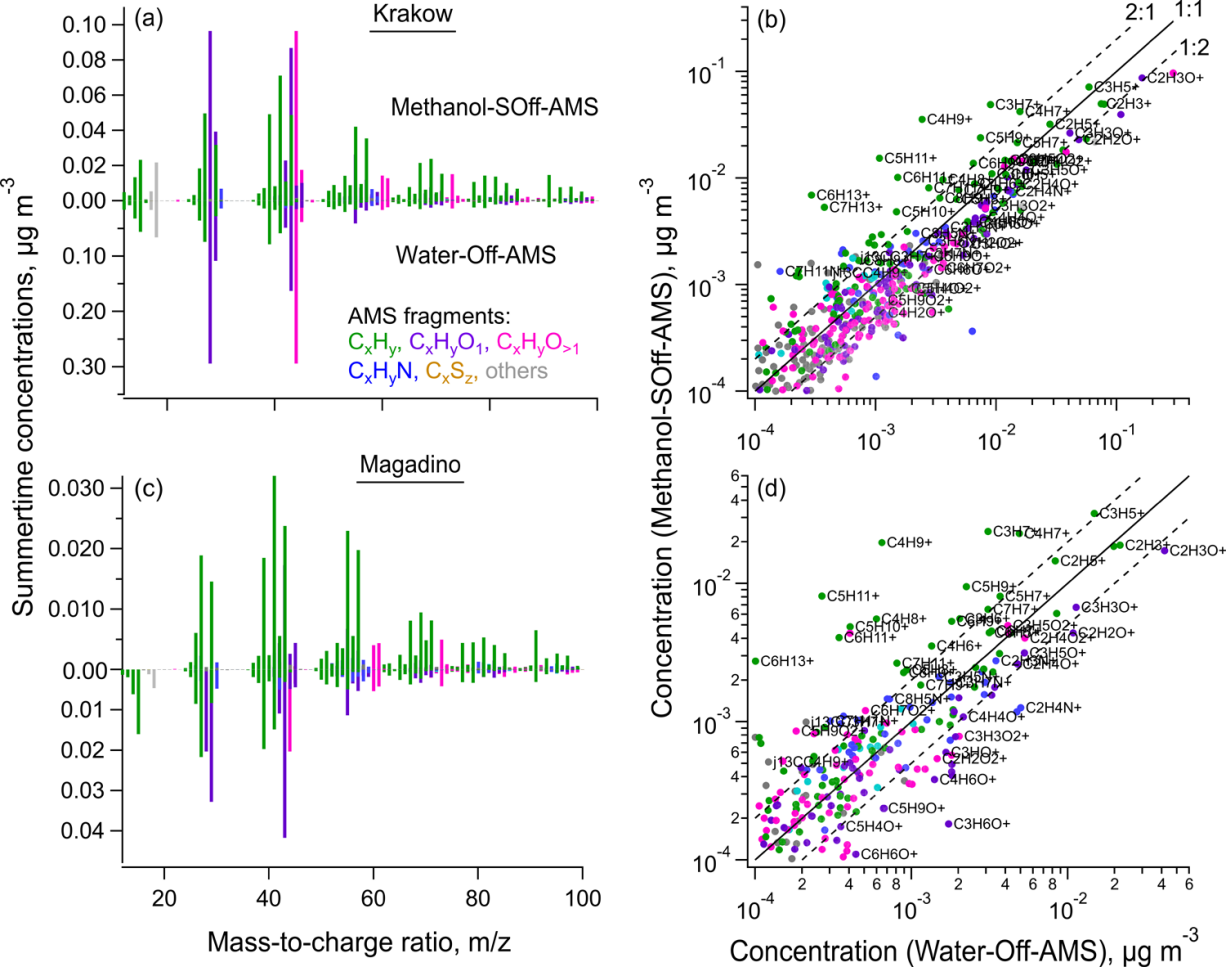
Summertime high-resolution mass spectra of coarse OA from Krakow and Magadino extracted in (a) methanol and (c) water. (b–d) Comparisons between normalized mass spectra of coarse OA from (b) Krakow and (d) Magadino extracted in methanol and water. CH_2_O^+^, CH_3_O^+^, and CH_4_O^+^ are blanked since those are solvent-related peaks (Figure S8).

In summary, SOff-AMS is a transferable analytical protocol that can be widely used to comprehensively characterize ambient OA from diverse areas including where in situ online measurements may not be feasible due to cost and logistical considerations. It can also help investigate the composition and sources of coarse and other larger particles that are typically not accessible via online AMS measurements.

## Supplementary Material



## Data Availability

The data for the main figures in this study is archived in the Zenodo repository and will be publicly available at 10.5281/zenodo.16880946 upon the publication of this article.

## Notes

Additional SOff-AMS analyses including calibrations; spectra intercomparisons and stability; measurements of water soluble, total, organic, and elemental carbon in aerosol samples; and OM/OC measurements and probability density distributions of OA factors across multiple European sites (PDF)

## ⊥

Institute of Climate and Energy Systems, ICE-3: Troposphere, Forschungszentrum Jülich GmbH, 52428 Jülich, Germany

## #

Datalystica Ltd., Parkstrasse 1, 5234 Villigen, Switzerland
