# Supplementary material for: Organic Solvents-Based Offline Aerosol Mass Spectrometry (SOff-AMS) for Comprehensive Chemical Characterization of Ambient Organic Aerosol
Source: Environ Sci Technol. 2025 Aug 19;59(34):18236–48. doi: 10.1021/acs.est.5c08949 (PMC12409874; doi:10.1021/acs.est.5c08949)
Supplement: Supplementary file 1 [file es5c08949_si_001.pdf]

Supplemental Information

Organic Solvents-Based Offline Aerosol Mass Spectrometry (SOff-AMS) for Comprehensive Chemical Characterization of Ambient Organic Aerosol

Peeyush Khare[1,¶,*], Abdul Aziz Kurdieh[1], Yufang Hao[1], Manousos-Ioannis Manousakas[1,2], Lubna Dada[1], Anna Tobler[1,β], Kristty Schneider-Beltran[1], Evangelia Diapouli[2], Alicja Skiba[3], Katarzyna Styszko[4], André S.H. Prévôt[1], Kaspar R. Daellenbach[1,*]

[1]PSI Center for Energy and Environmental Sciences, Paul Scherrer Institute, 5232 Villigen, Switzerland
[2]ENRACT, Institute of Nuclear & Radiological Sciences and Technology, Energy & Safety, N.C.S.R. "Demokritos", Ag. Paraskevi 15310, Greece
[3]AGH University of Krakow, Faculty of Physics and Applied Computer Science, 30-059 Krakow, Poland
[4]AGH University of Krakow, Faculty of Energy and Fuels, 30-059 Krakow, Poland

[¶]Now at: Institute of Climate and Energy Systems, ICE-3: Troposphere, Forschungszentrum Jülich GmbH, 52428 Jülich, Germany
[β]Now at: Datalystica Ltd., Parkstrasse 1, 5234 Villigen, Switzerland

[*]All correspondence shall be addressed to: Peeyush Khare (p.khare@fz-juelich.de) and Kaspar R. Daellenbach (kaspar.daellenbach@psi.ch)

Summary:
Total pages: 28
Total figures: 26

Table of contents

**Fig. S1**	Mass calibration curves for ammonium -nitrate and -sulfate standards.
**Fig. S2**	HR spectra for levoglucosan standard measured in methanol, acetone and water.
**Fig. S3**	Deviations in levoglucosan spectra relative to the best estimate in different solvents.
**Fig. S4**	Variations in contributions of select ions relative to $C_2H_4O_2^+$ in levoglucosan spectra for different solvents.
**Fig. S5**	Variations in the OM:OC ratios of the levoglucosan spectra relative to the best estimate.
**Fig. S6**	Solvent bias in the levoglucosan aerosol spectra aerosolized at different concentrations.
**Fig. S7**	Interference of solvent organics in inorganic aerosol measurements.
**Fig. S8**	Mass spectra of spiked methanol, acetone and water.
**Fig. S9**	SOff-AMS spectra of filter blanks compared with spiked solvents.
**Fig. S10**	Probability density distributions of OA factor contributions in Krakow, Magadino and 17 other European sites.
**Fig. S11**	Water solubilities of fine PM OA samples collected from Krakow and Magadino.
**Fig. S12**	Total carbon, organic and elemental carbon concentrations for the analyzed filters from Krakow and Magadino.
**Fig. S13**	SOff-AMS and water-Off-AMS HR spectra of winter- and summer-time fine OA from Krakow and Magadino.
**Fig. S14**	SOff-AMS and water-Off-AMS HR spectra of winter- and summertime PM_{10} OA from Krakow and Magadino.
**Fig. S15**	Unit mass resolution SOff-AMS spectra of PM_{10}, fine OA samples.
**Fig. S16**	The OM:OC ratios for Krakow and Magadino samples extracted in methanol, acetone and water.
**Fig. S17**	Enhanced contributions of key aerosol mass fragments in acetone-SOff-AMS spectra.
**Fig. S18**	HR peaks fits showing reduced prevalence of $CH_3SO_2^+$ fragment signal in organic solvent extracts.
**Fig. S19**	Difference spectra between methanol and water extracts of the analyzed samples from Krakow and Magadino.
**Fig. S20**	Cosine angle similarities between high-resolution mass spectra (m/z 12 – 152).
**Fig. S21**	Cosine angle similarities between unit mass resolution mass spectra (m/z 12 – 467).
**Fig. S22**	Krakow online Q-ACSM compared with acetone-SOff-AMS for winter and summertime fine OA.
**Fig. S23**	Scatter plots comparison fine OA mass spectra obtained from acetone-SOff-AMS and online Q-ACSM
**Fig. S24**	Krakow online Q-ACSM compared with water-Off-AMS for winter and summertime fine OA.
**Fig. S25**	Stability of the SOff-AMS spectra of sample extracts measured before and after 36 hours.
**Fig. S26**	Summertime high-resolution mass of coarse OA from Krakow and Magadino.

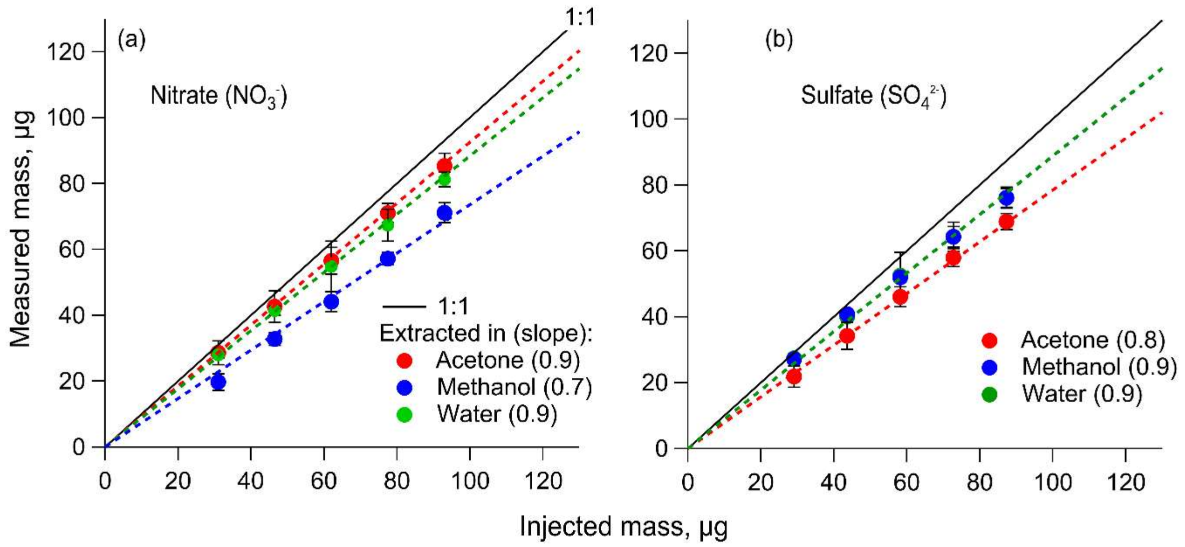

Figure S1. Mass calibration curves for ammonium -nitrate and -sulfate standards.

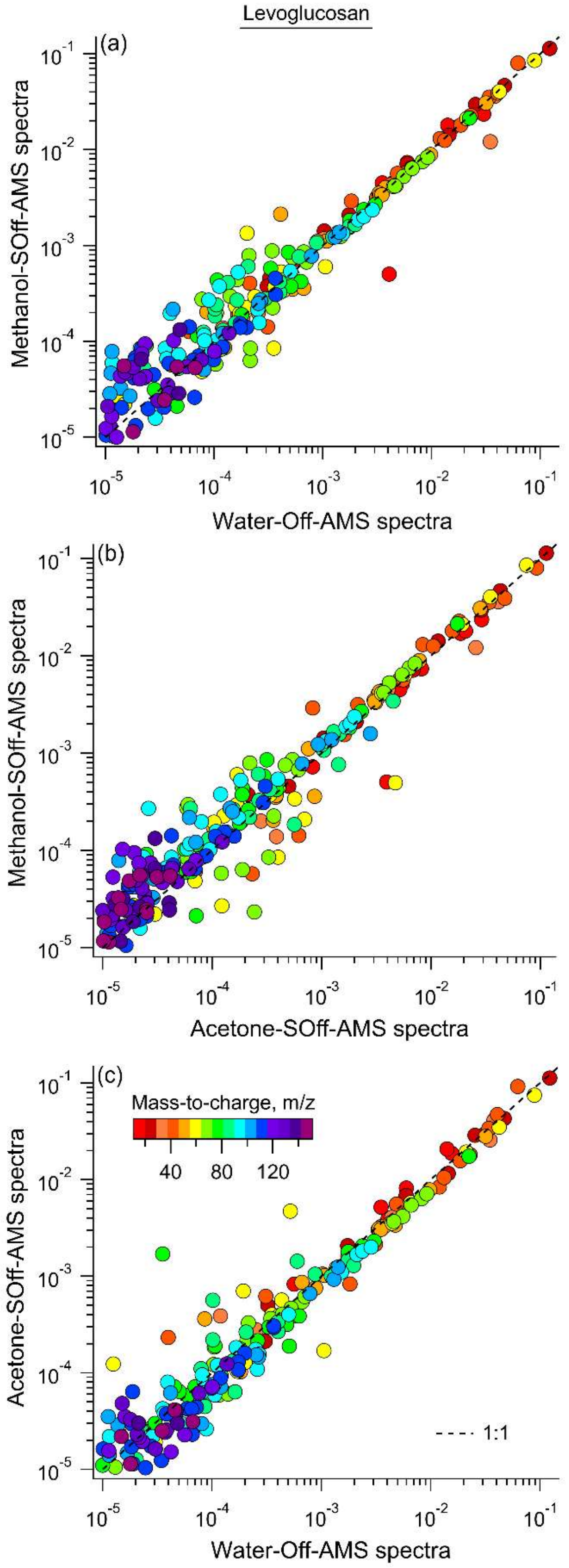

Figure S2. Comparison of HR spectra for Levoglucosan standard measured in methanol, acetone and water. Methanol and acetone-based spectra show strongest similarities across different AMS fragment groups.

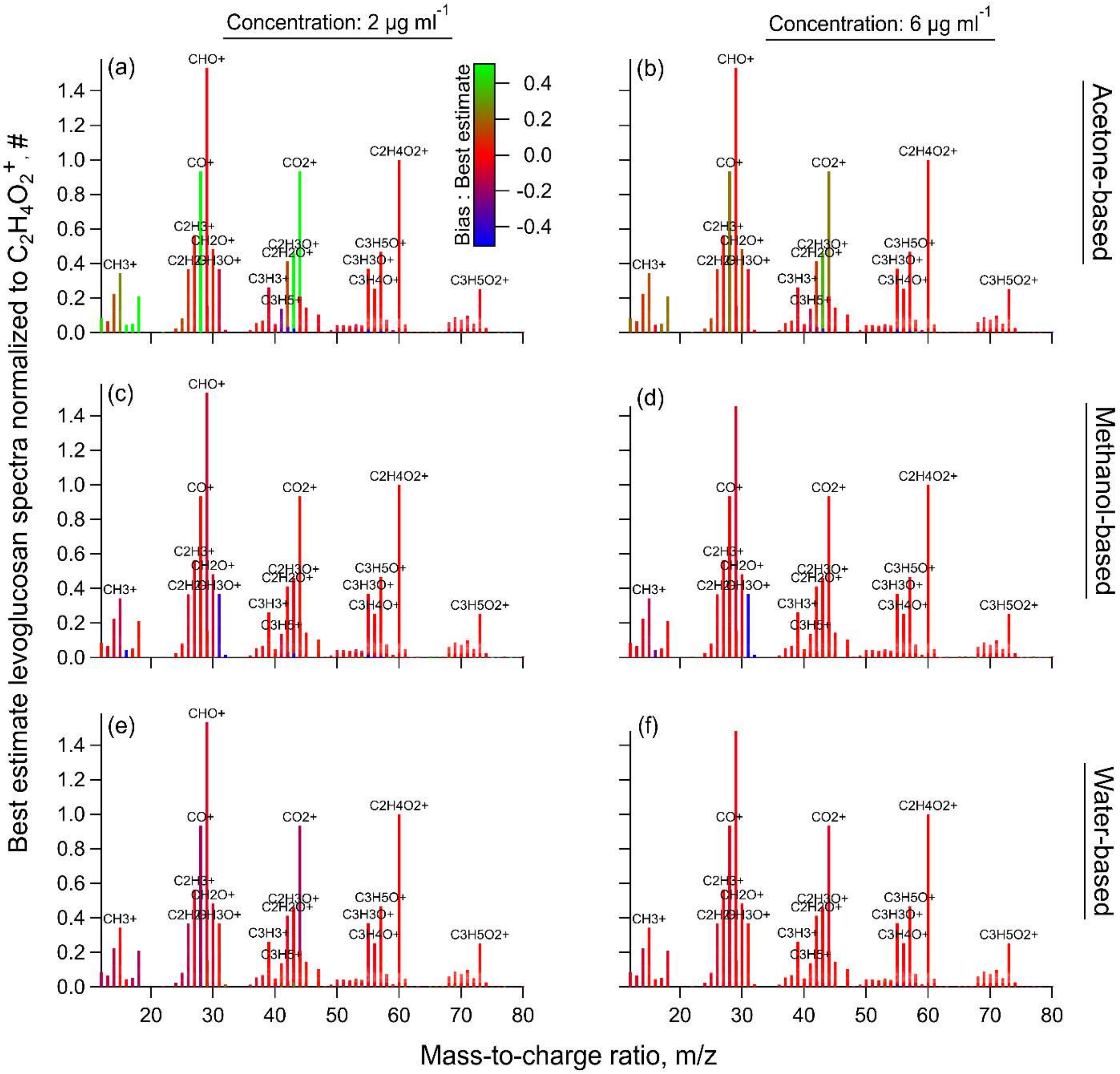

Figure S3. (a-f) The best estimate of levoglucosan spectra derived as the average spectra of the highest concentration of levoglucosan (6 µg ml^{-1}) in acetone, methanol and water. The color codes in each panel represent deviations relative to the best estimate in spectra obtained from the lowest (left column: 2 µg ml^{-1}) and highest (right column: 6 µg ml^{-1}) levoglucosan concentrations prepared in (a,b) acetone, (c,d) methanol and (e,f) water as solvents. Note: For these comparisons, all spectra were normalized to $C_2H_4O_2^+$.

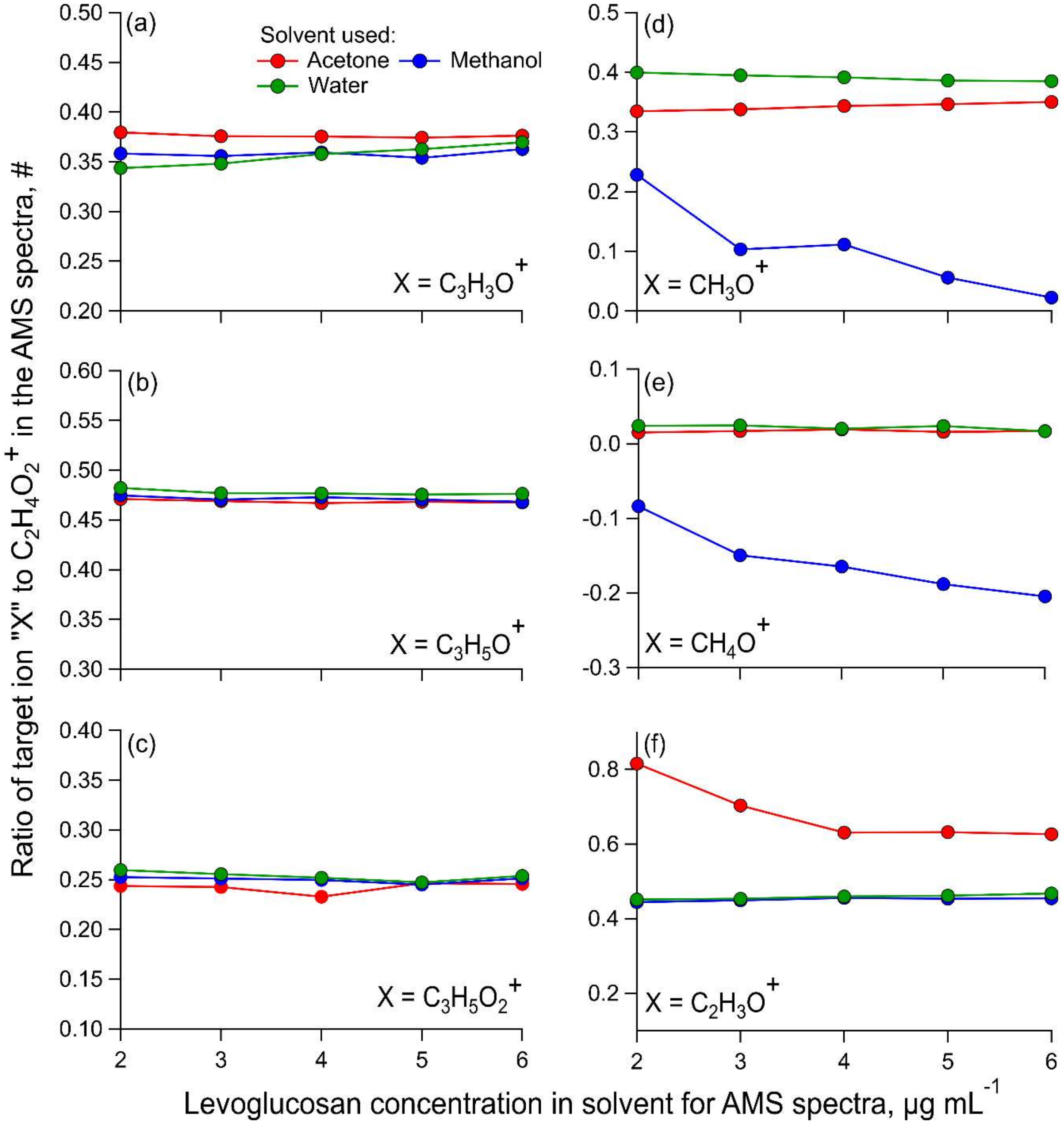

Figure S4. Variations in the contributions of target ion "X" relative to $C_2H_4O_2^+$ in aerosol spectra obtained for different concentrations of levoglucosan (2 – 6 µg ml^{-1}) dissolved in acetone, methanol and water.

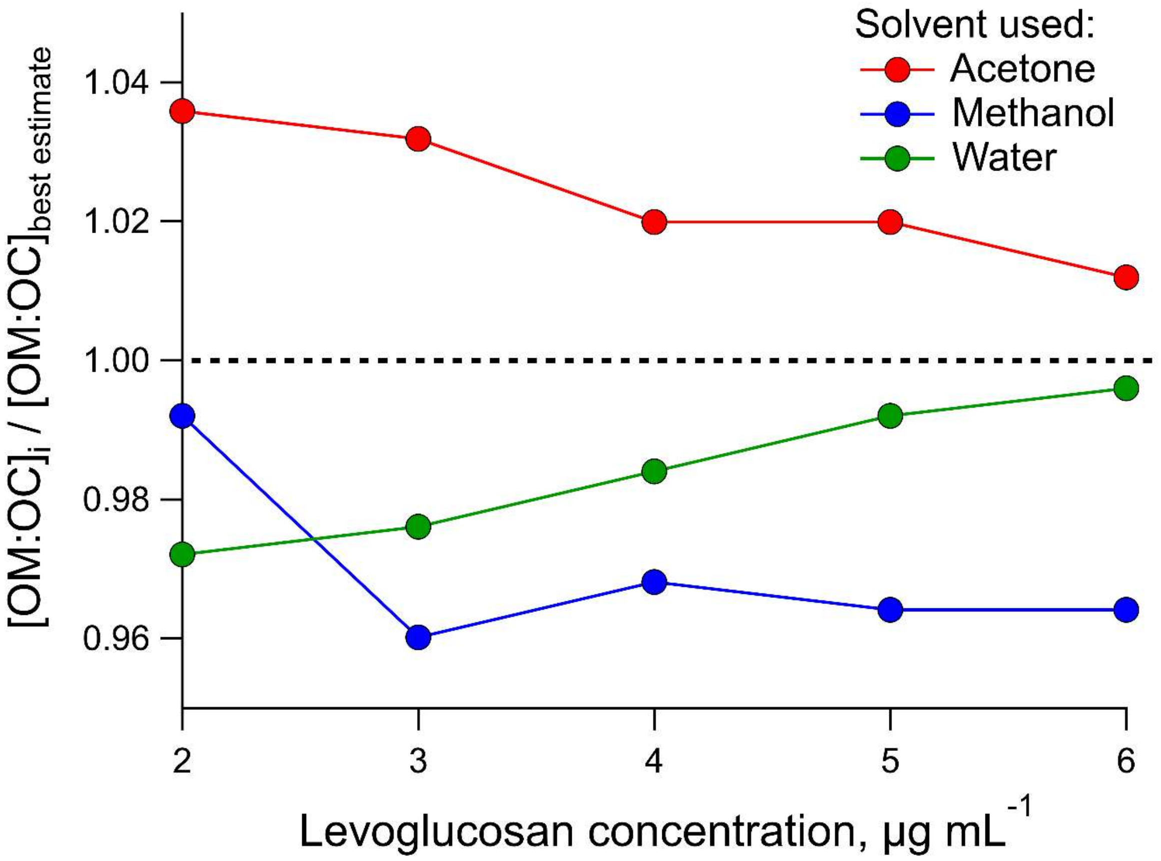

Figure S5. Variations in the OM:OC ratio of levoglucosan aerosol spectra relative to the best estimate of levoglucosan aerosol spectra for different concentrations, "i" (2 – 6 µg ml^{-1}), of levoglucosan dissolved in acetone, methanol and water. The dashed line represents 100% agreement with the OM:OC ratio of the best estimate.

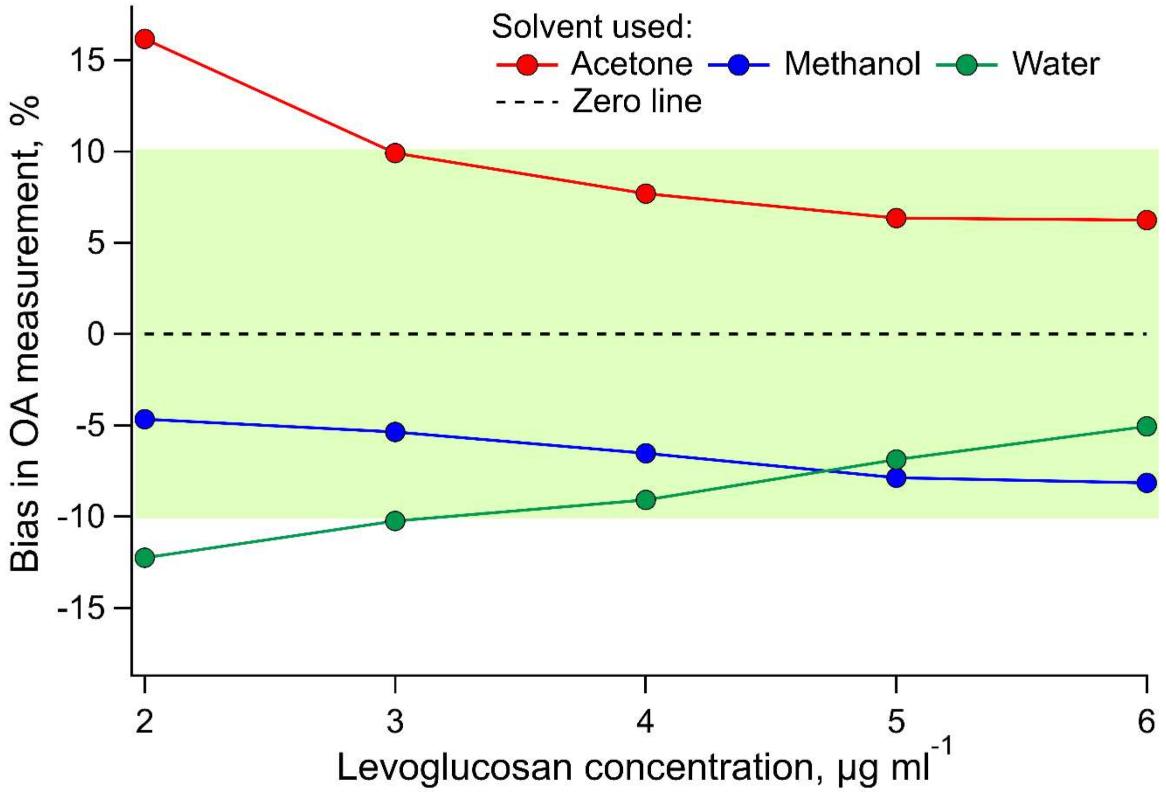

Figure S6. Solvent bias in the mass spectra of levoglucosan aerosol extracted at five different concentrations in acetone, methanol and water. The area shaded in green indicates bias under 10%.

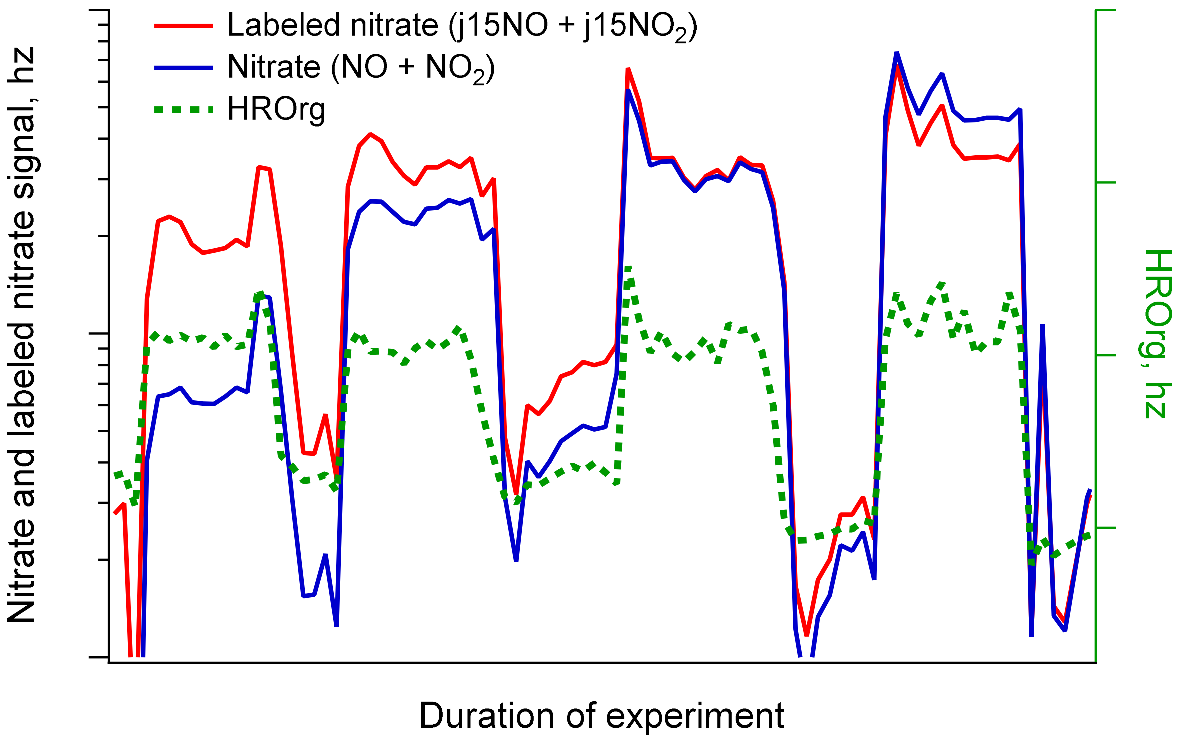

Figure S7. (a) HROrg, NO^+ and NO_2^+ fragment signal frequencies measured by the ToF-AMS.

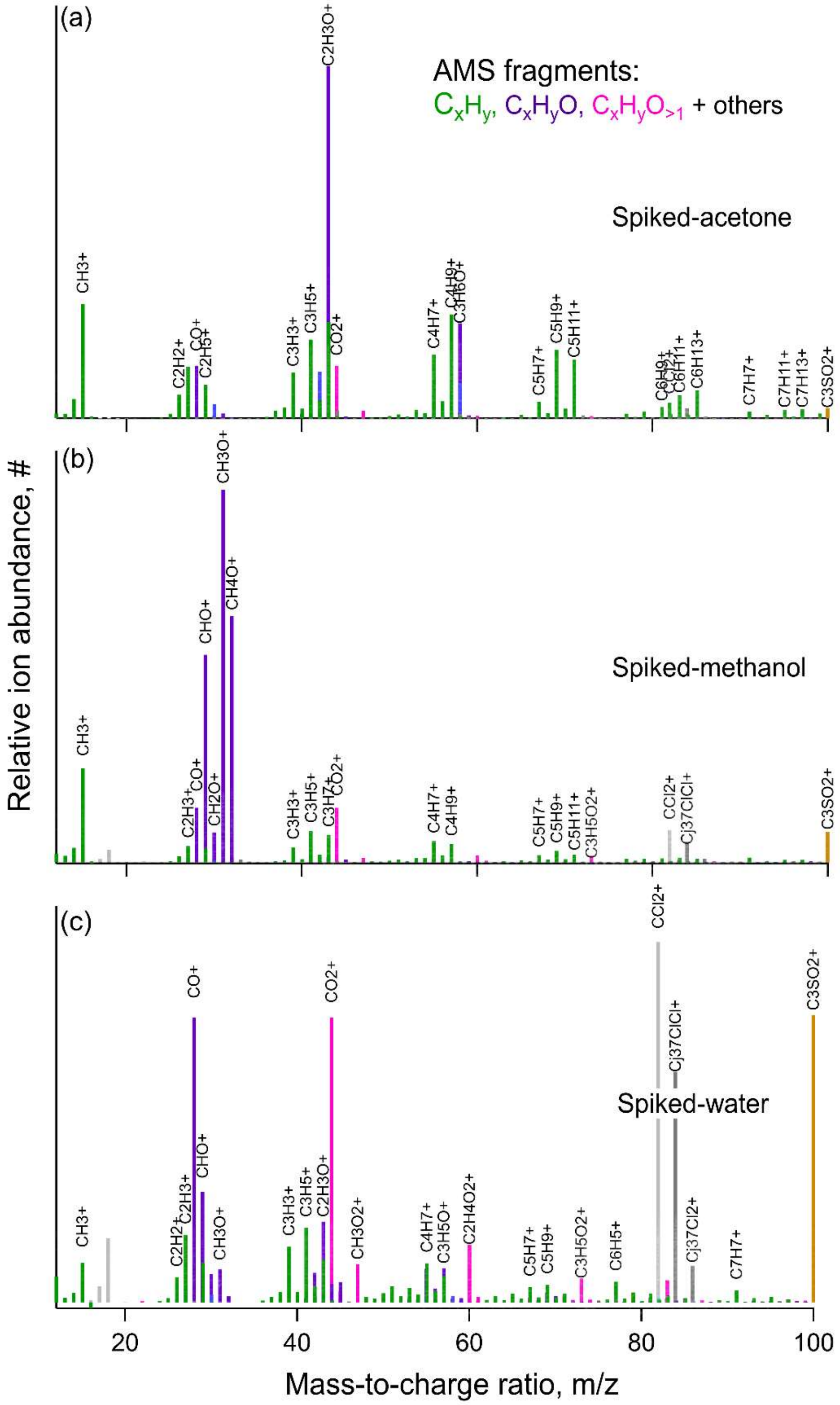

Figure S8. Mass spectra of (a) acetone, (b) methanol and (c) water spiked with labelled inorganic standards and measured via ToF-AMS.

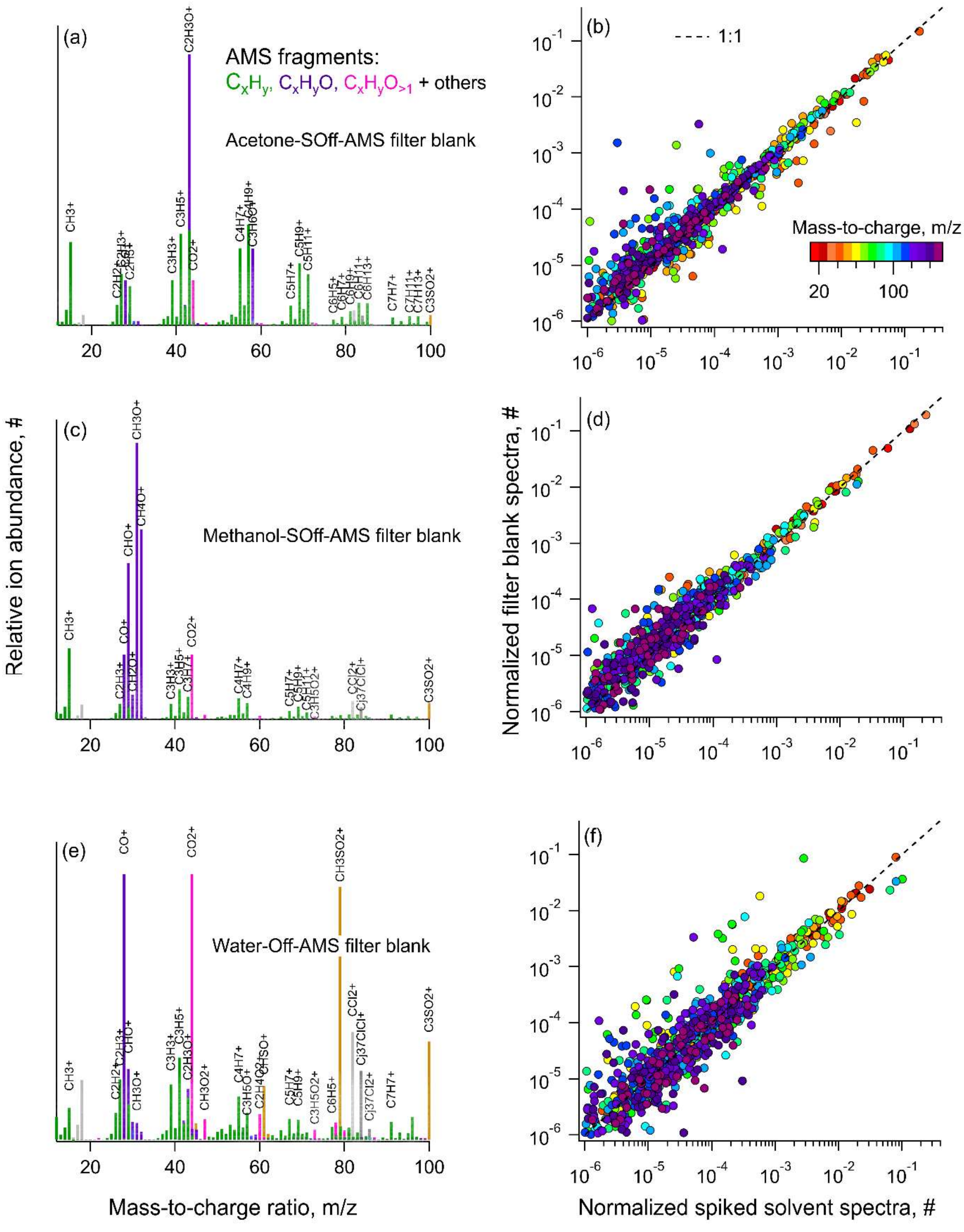

Figure S9. SOff-AMS spectra of filter blanks extracted in (a) acetone, (c) methanol and (e) water, compared with spiked (b) acetone, (d) methanol and (f) water solvents.

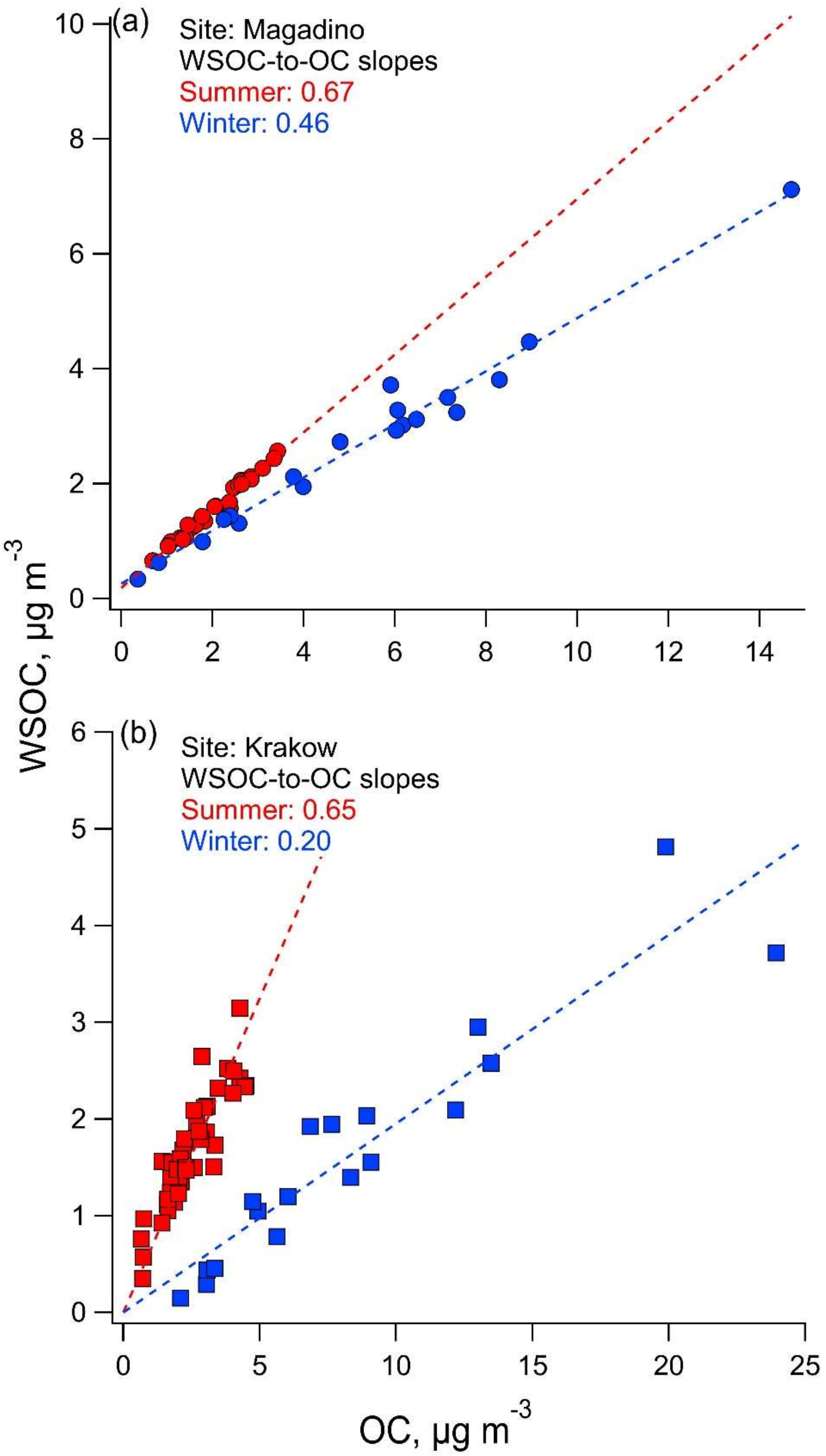

Figure S10. Water solubilities of summer- and winter-time fine PM OA samples collected from (a) Magadino and (b) Krakow. The dashed lines indicate slopes of the WSOC vs. OC distributions. The slopes correspond to the water solubilities of samples selected for this study as shown in figure 3.

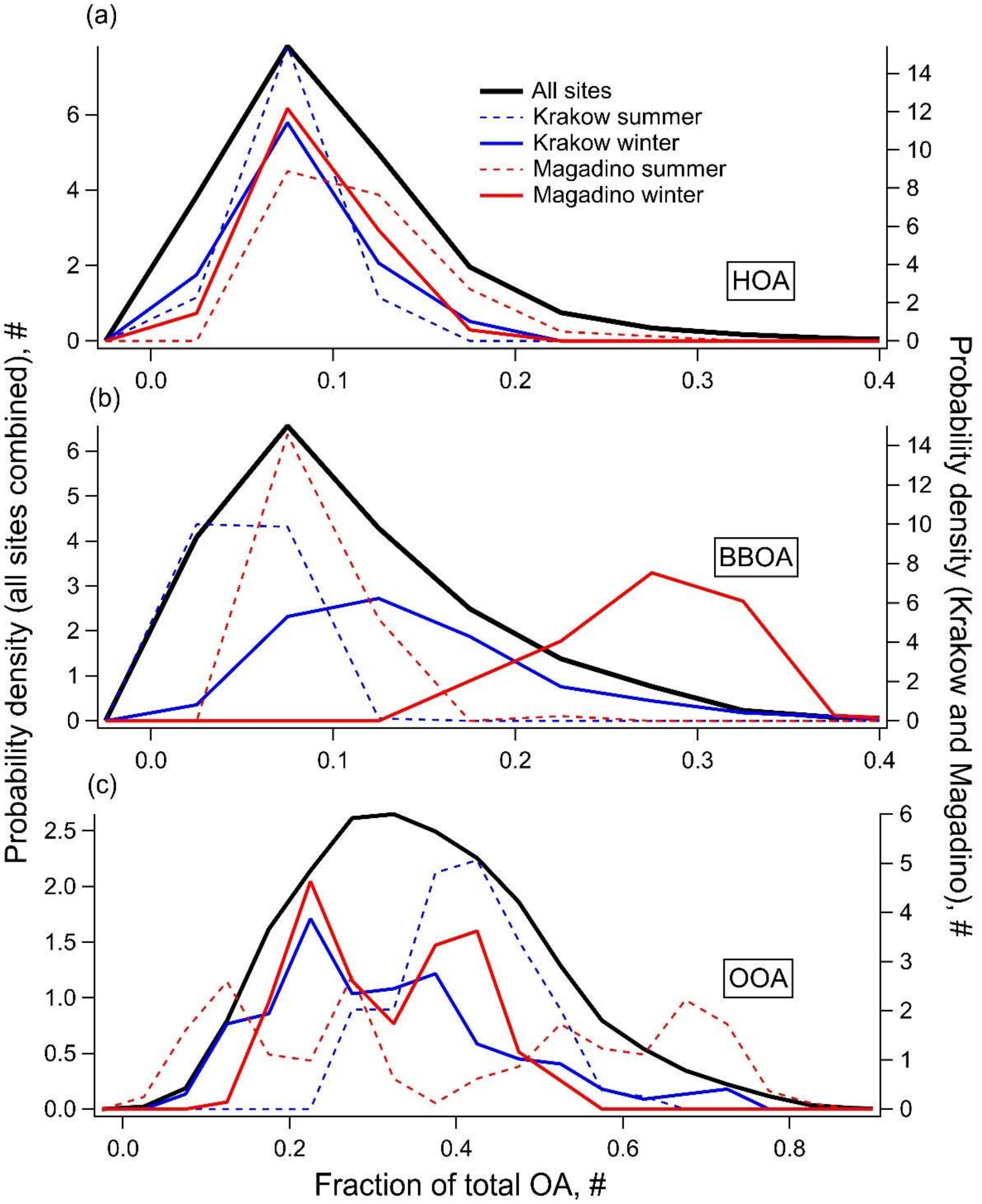

Figure S11. Probability density distributions of the contributions of (a) HOA, (b) BBOA and (c) OOA factors to fine OA compared between summer- and winter-time OA in Krakow and Magadino and 17 sites across Europe (all sites).[1] Note: The BBOA factor also includes coal-combustion (CCOA) emissions to fine OA. OOA includes both less- and more-oxygenated OA (LO-OOA and MO-OOA) contributions.

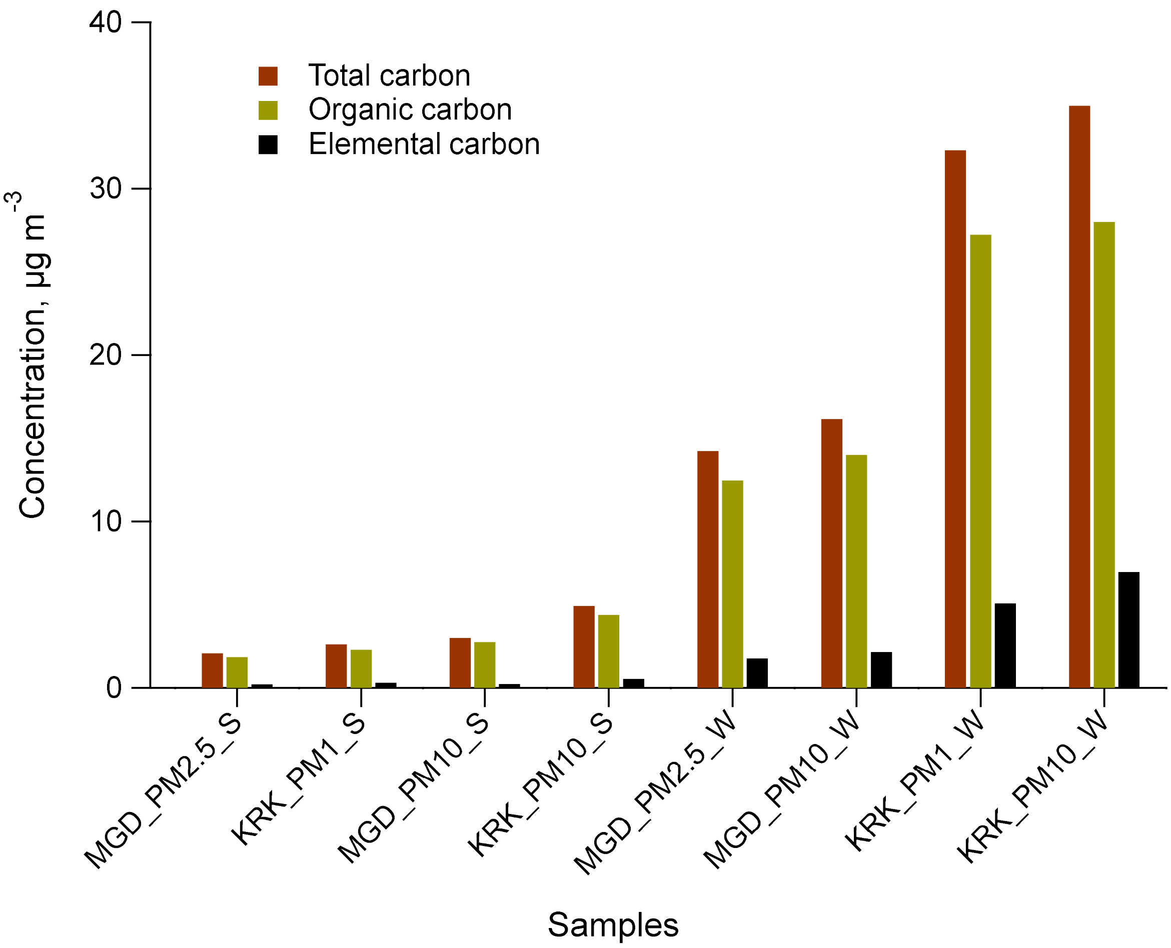

Figure S12. Total carbon, organic and elemental carbon concentrations for the analyzed filters from Krakow and Magadino.

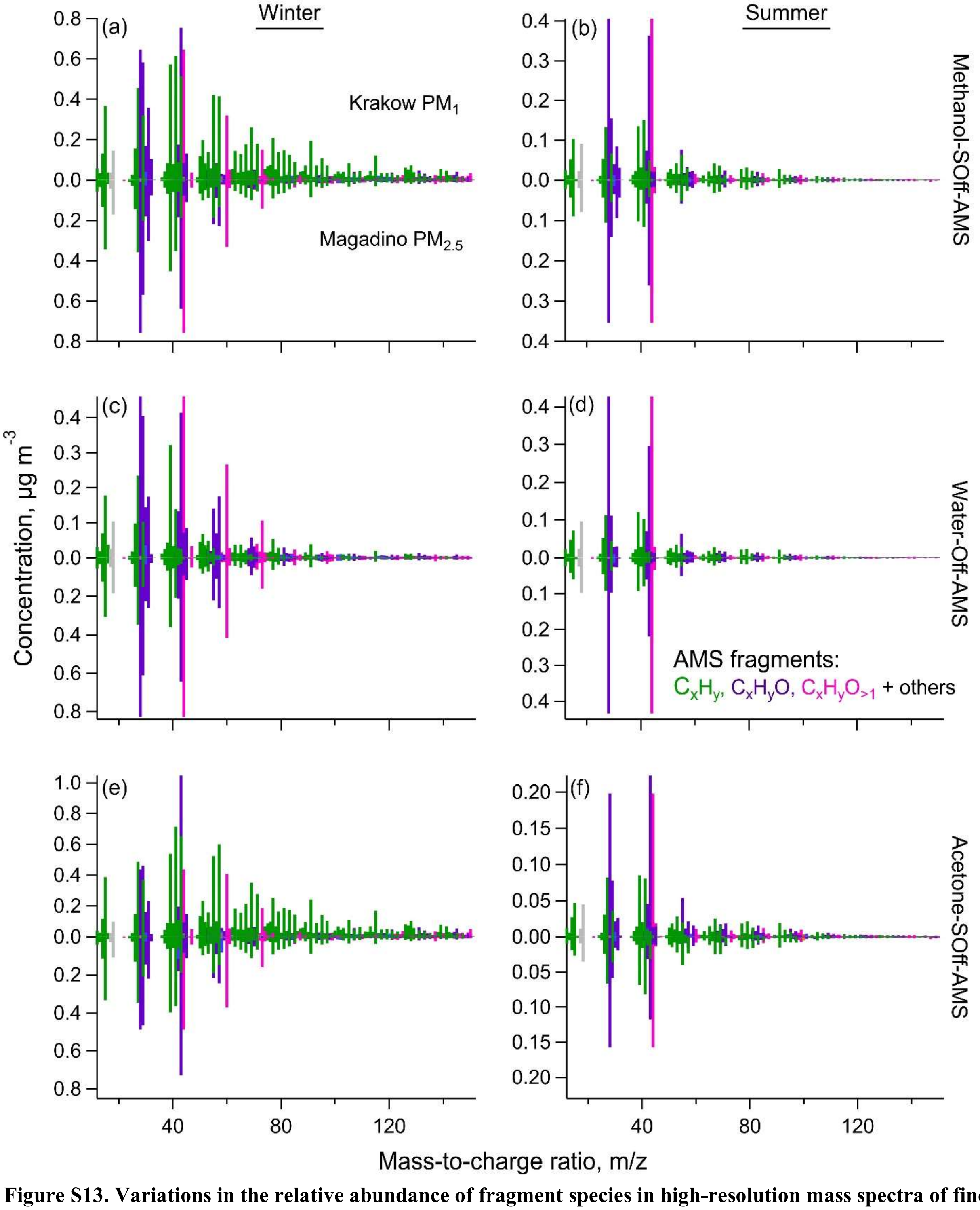

Figure S13. Variations in the relative abundance of fragment species in high-resolution mass spectra of fine OA from Krakow and Magadino. The panels (a-d) show each panel with Krakow (top y-axis) and Magadino (bottom y-axis) spectra separated by seasons (a,c: winter; b,d: summer) and extraction solvent (a,b: methanol, c,d: water). The inset figures in each panel show correlations between the spectra color-coded by the m/z values.

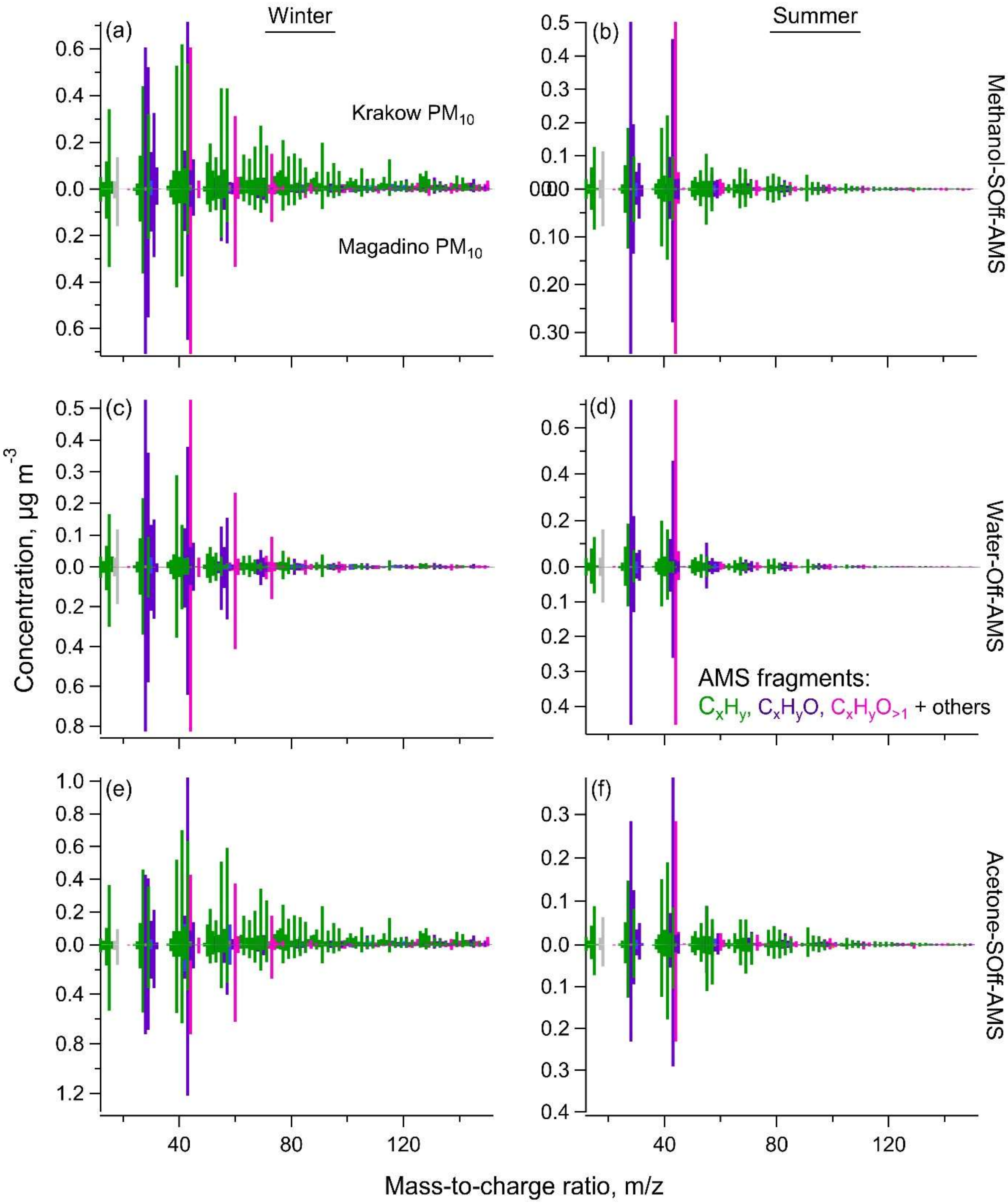

Figure S14. Variations in the relative abundance of fragment species in high-resolution mass spectra of PM_{10} OA from Krakow and Magadino. The panels (a-d) show each panel with Krakow (top y-axis) and Magadino (bottom y-axis) spectra separated by seasons (a,c: winter; b,d: summer) and extraction solvent (a,b: methanol, c,d: water). The inset figures in each panel show correlations between the spectra color-coded by the m/z values.

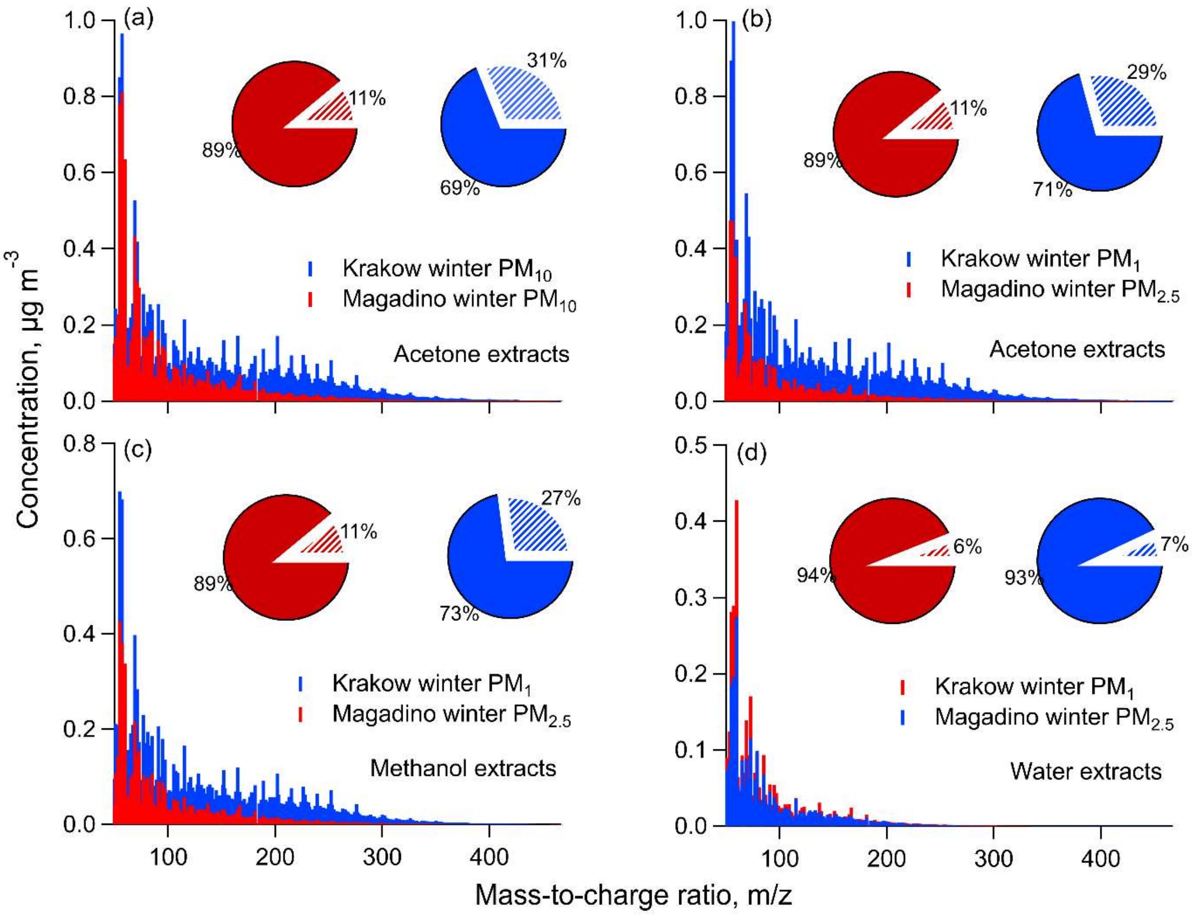

Figure S15. Unit mass resolution SOff-AMS spectra of PM filters from Krakow and Magadino including PM_{10}, PM_1 and $PM_{2.5}$ samples extracted in (a,b) acetone, (c) methanol and (d) water.

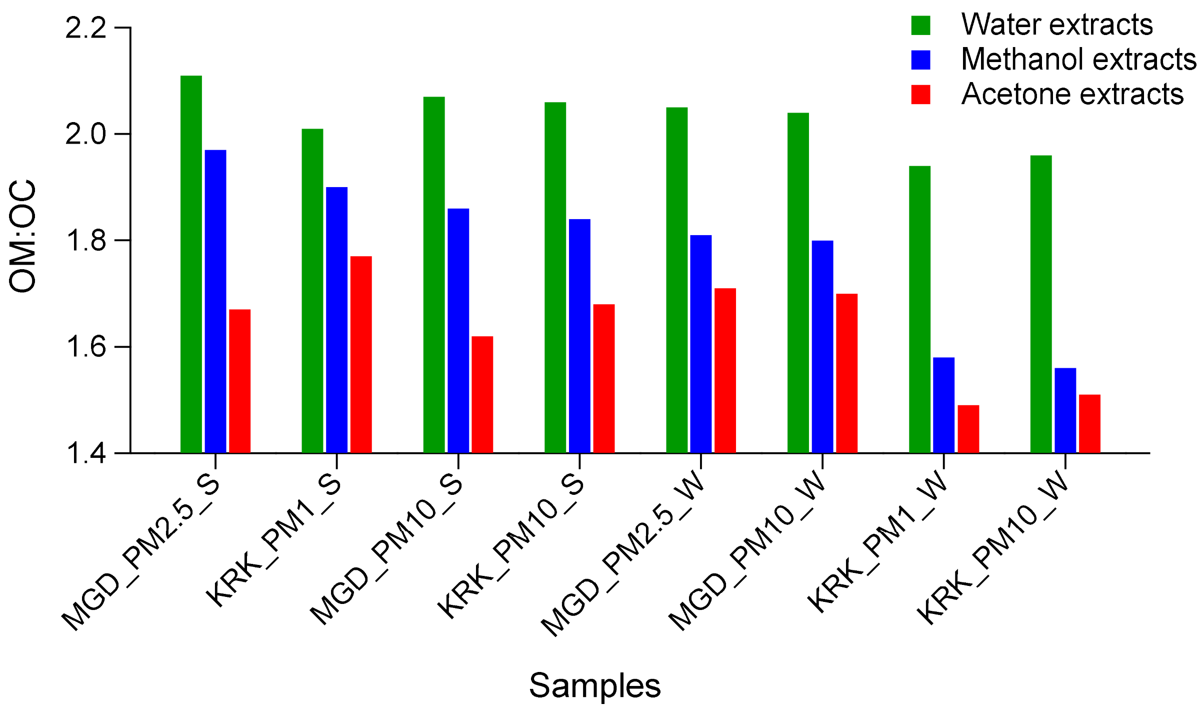

Figure S16. The OM:OC ratios for Krakow and Magadino samples extracted in methanol, acetone and water measured via AMS.

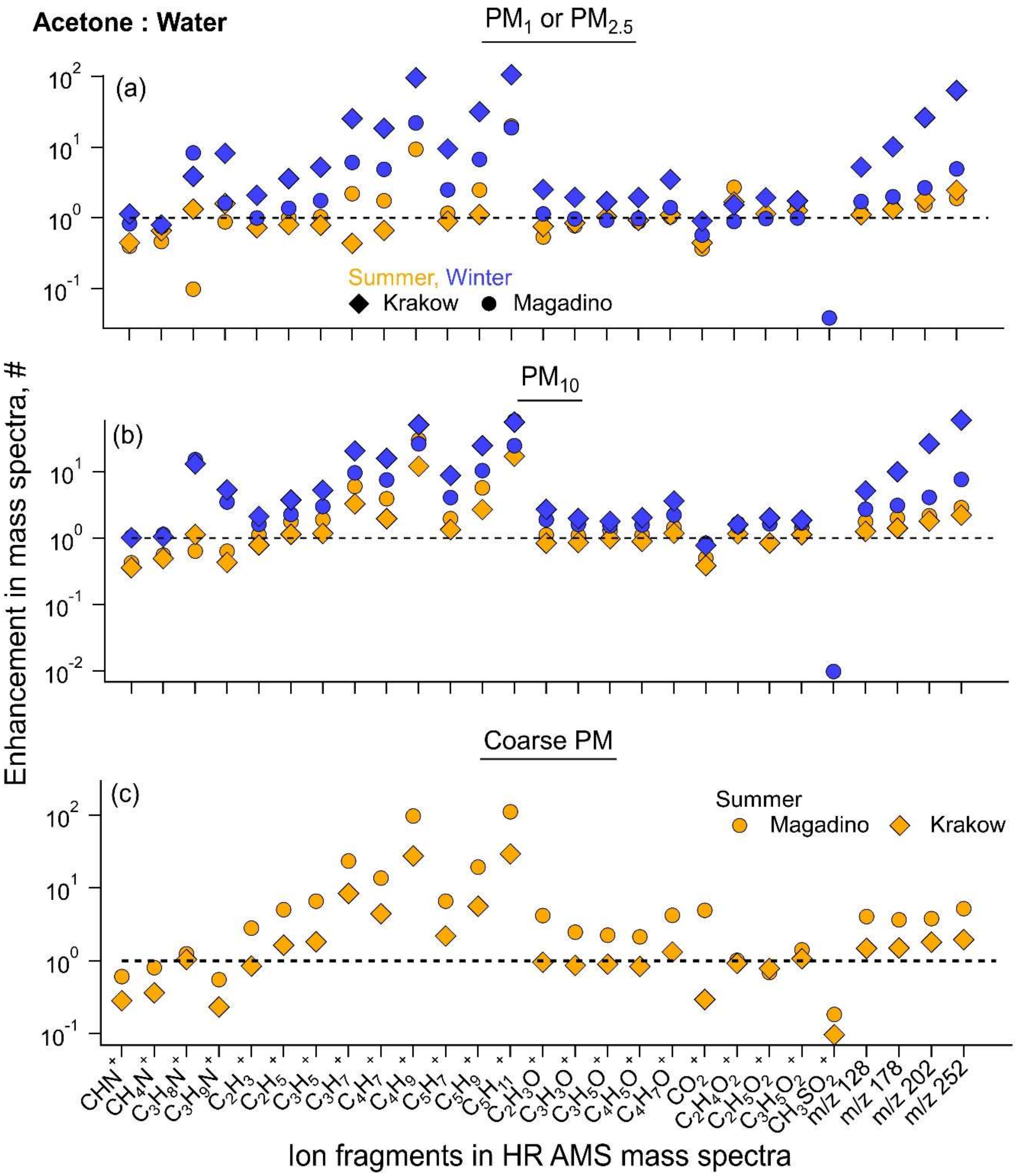

Figure S17. Enhanced contributions of key aerosol mass fragments to (a) fine, (b) PM10 and (c) coarse OA spectra in acetone-SOff-AMS relative to water-Off-AMS.

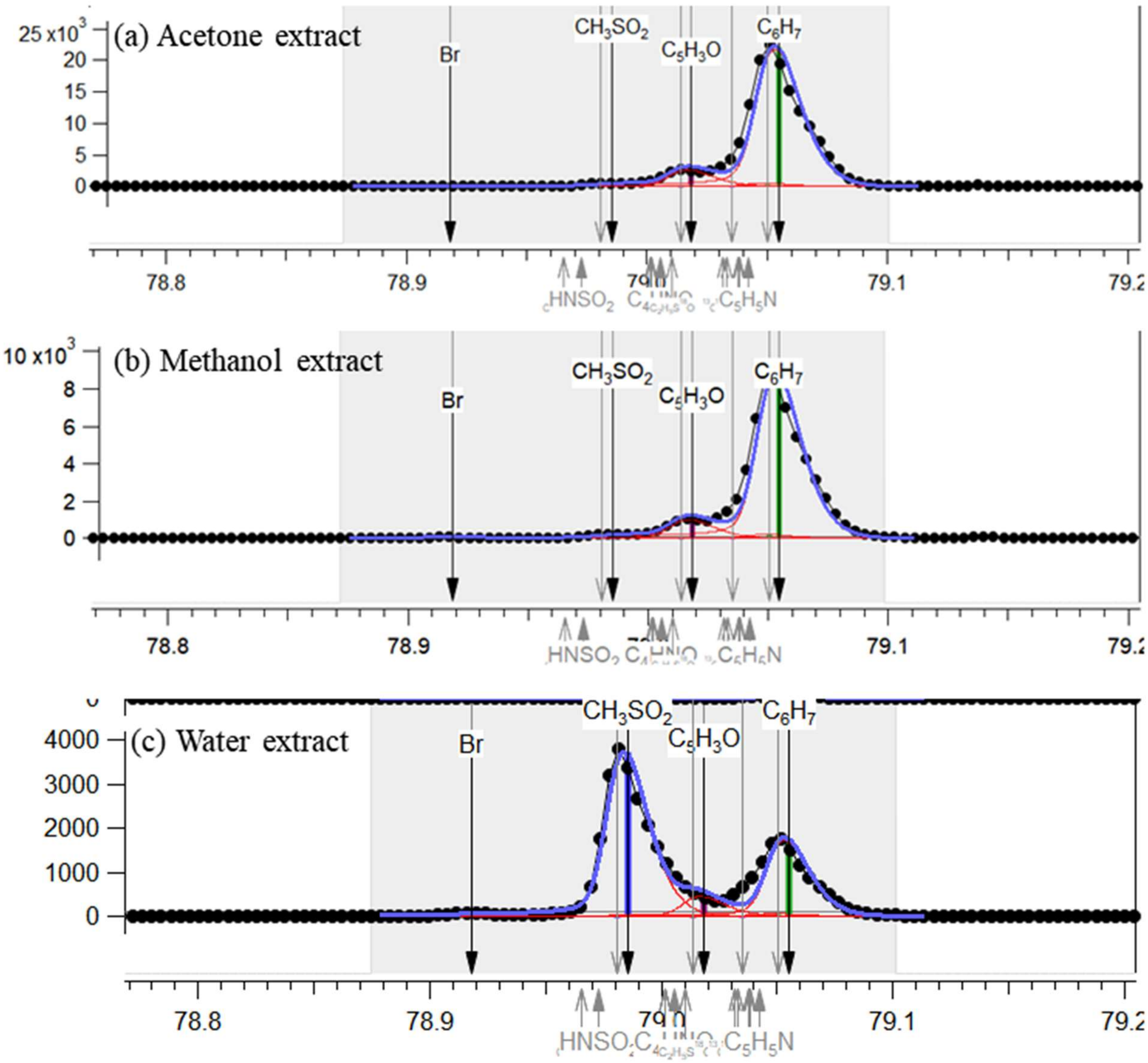

Figure S18. High-resolution peak fits to the ToF-AMS spectra showing reduced prevalence of $CH_3SO_2^+$ fragment in Krakow PM_{10} winter sample extracted in (a) acetone and (b) methanol relative to (c) water.

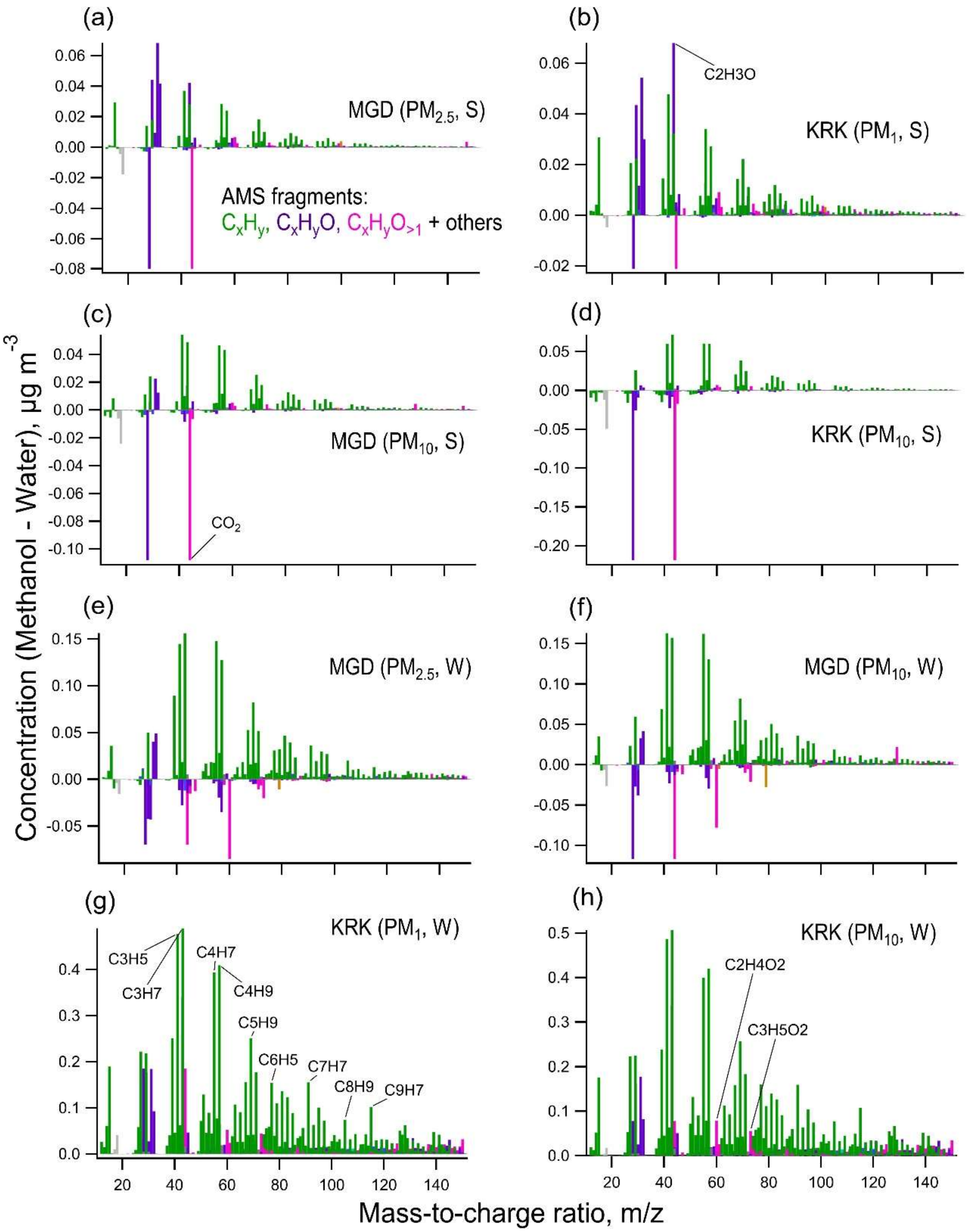

Figure S19. Difference spectra between methanol and water extracts for the analyzed filter samples. Key hydrocarbon and oxygenated fragments are labelled in panels b, c, g and h.

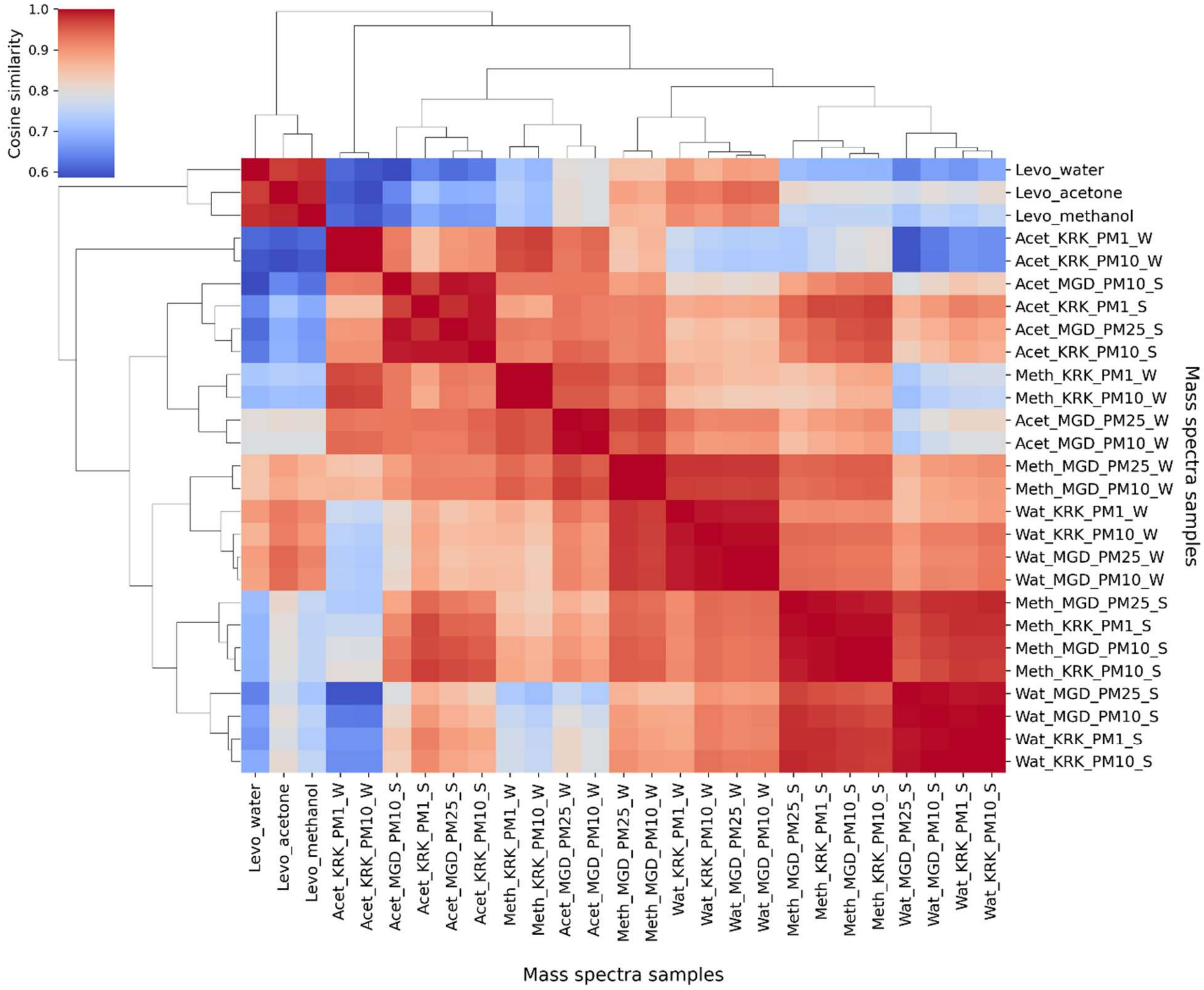

Figure S20. Cosine angle similarities between high-resolution mass spectra (m/z 12 – 152) of filter samples and levoglucosan standard OA aerosolized in all three solvents. Note: The term "Acet_KRK_PM1_W" indicates the Krakow (KRK) PM_1 filter sample collected in winter (W) and extracted in acetone (Acet). Similarly, Magadino (MGD), summer (S), meth (methanol) and wat (water).

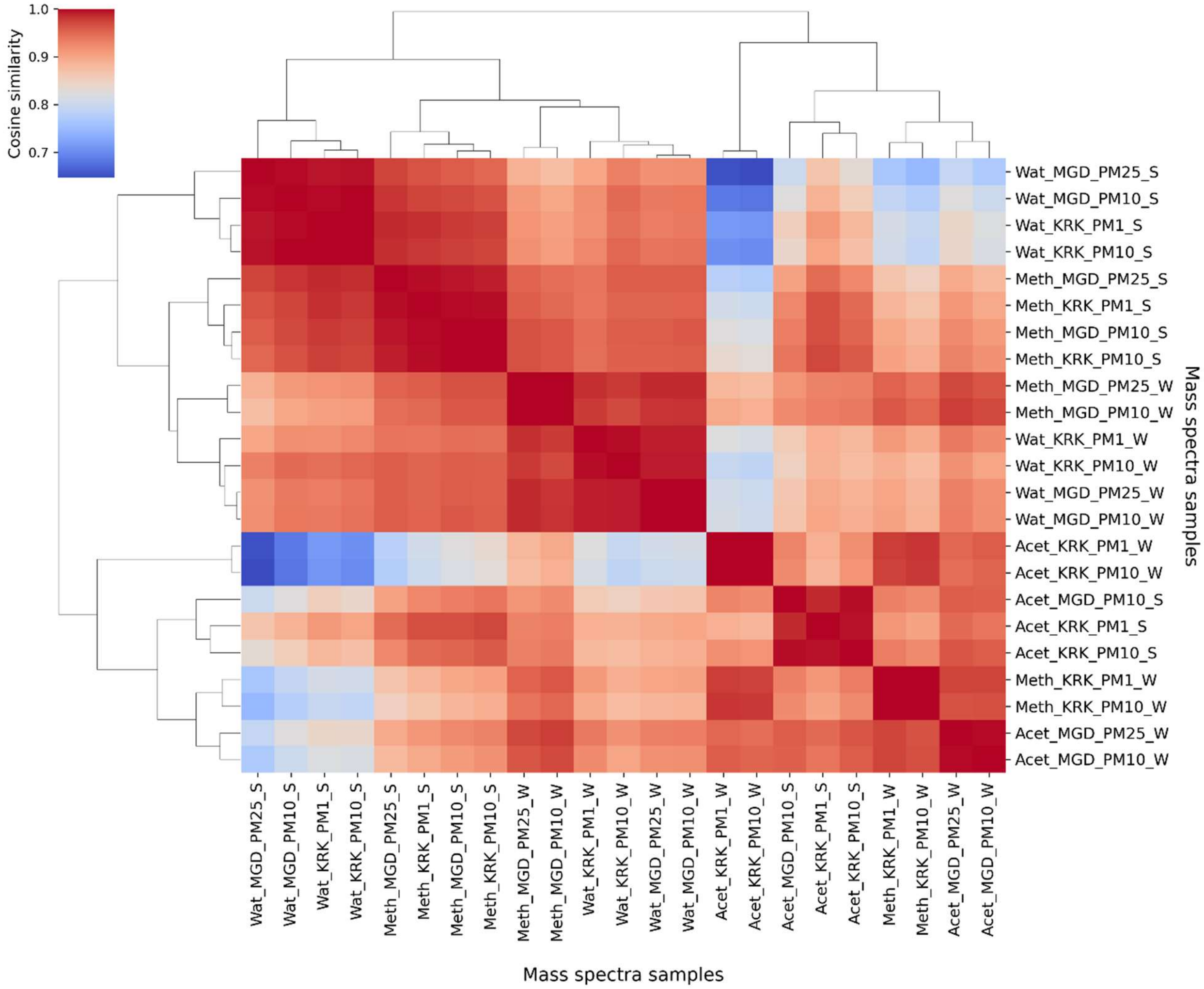

Figure S21. Cosine angle similarities between unit mass resolution spectra (m/z 12 – 467) of filter samples aerosolized in all three solvents. Note: The sample naming convention is described in the caption of figure S17.

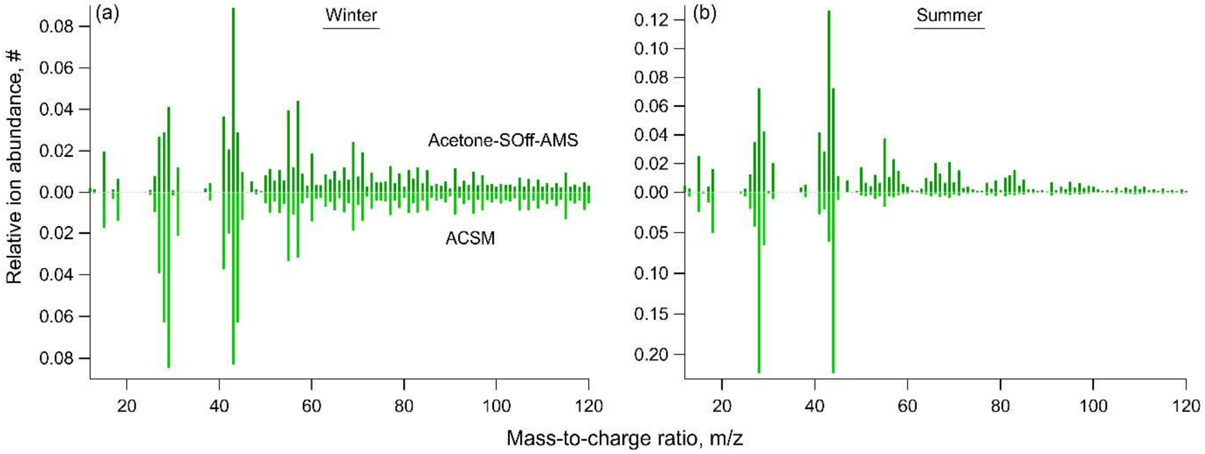

Figure S22. Fine OA mass spectra obtained from acetone-SOff-AMS and online Q-ACSM measurements in Krakow for (a) summer and (b) winter periods. Note: The ACSM spectra were averaged over the filter sample collection period and both spectra were normalized to 1 prior to comparison.

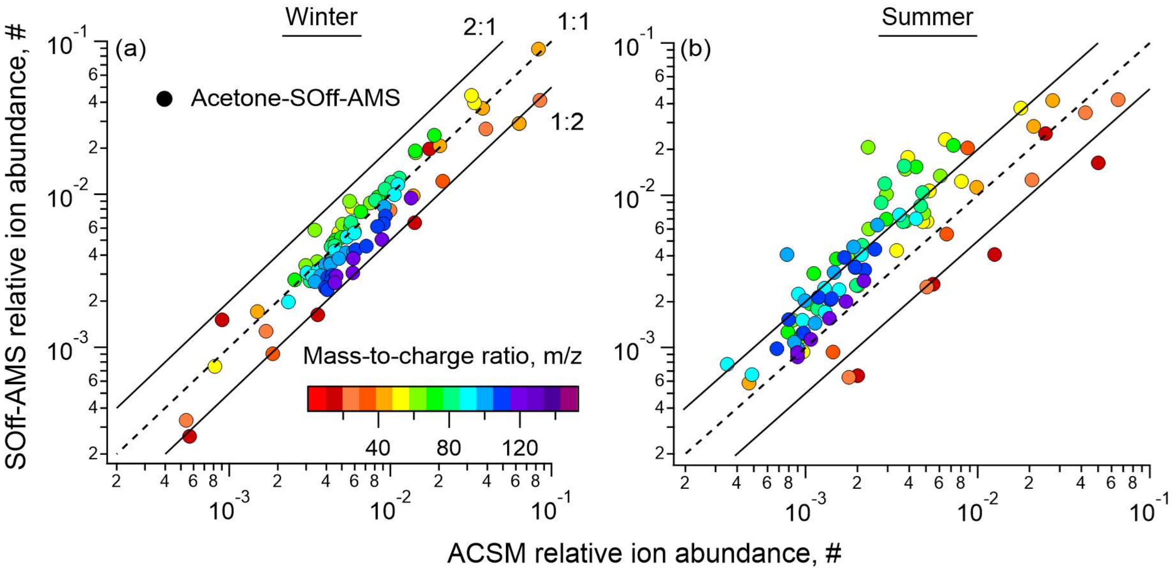

Figure S23. Scatter plots comparison fine OA mass spectra obtained from acetone-SOff-AMS and online Q-ACSM measurements in Krakow for (a) summer and (b) winter periods. Note: The ACSM spectra were averaged over the filter sample collection period. Both mass spectra were normalized to 1 prior to comparison.

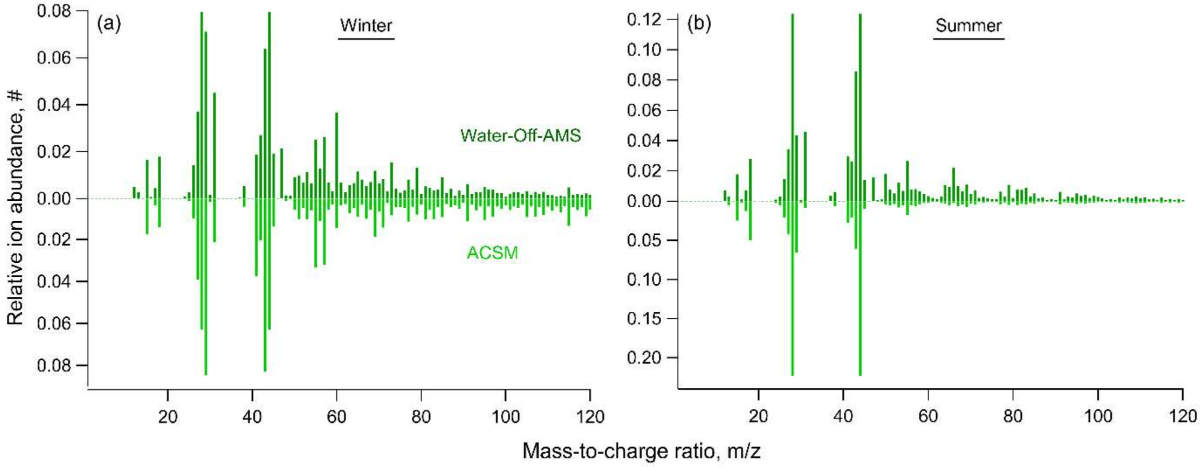

Figure S24. Fine OA mass spectra obtained from water-Off-AMS and online Q-ACSM measurements in Krakow for (a) summer and (b) winter periods. Note: The ACSM spectra were averaged over the filter sample collection period and both spectra were normalized to 1 prior to comparison.

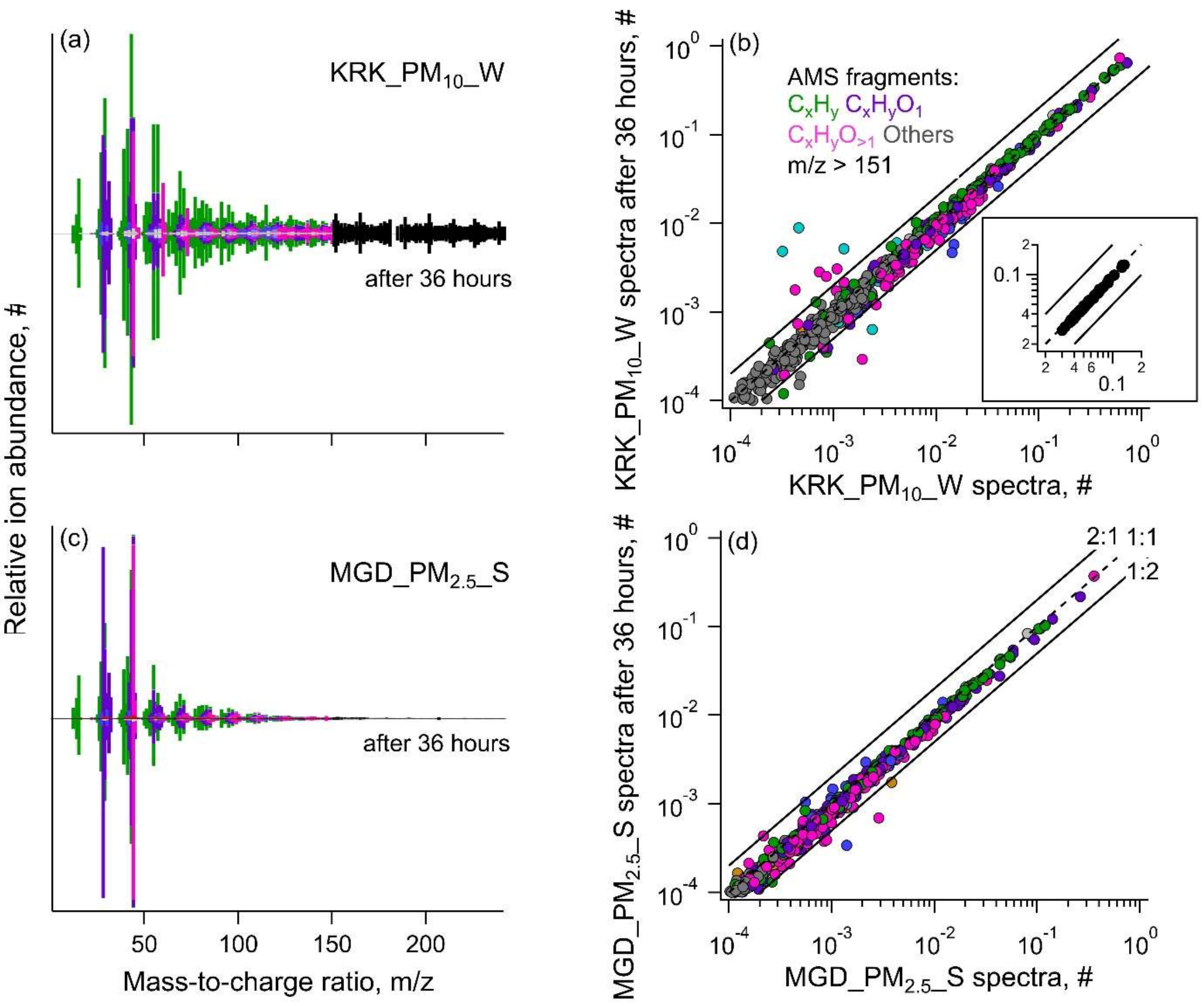

Figure S25. Stability of methanol-SOff-AMS spectra for (a,b) wintertime Krakow PM_{10} (KRK_PM_{10}_W) and (c,d) summertime Magadino $PM_{2.5}$ (MGD_$PM_{2.5}$_S) sample extracts assessed via repeat analyses before (top spectra in a,c) and after 36 hours of storage in dark at 4ºC (bottom spectra in a,c). The inset in (b) shows comparison of the UMR spectra for m/z > 150.

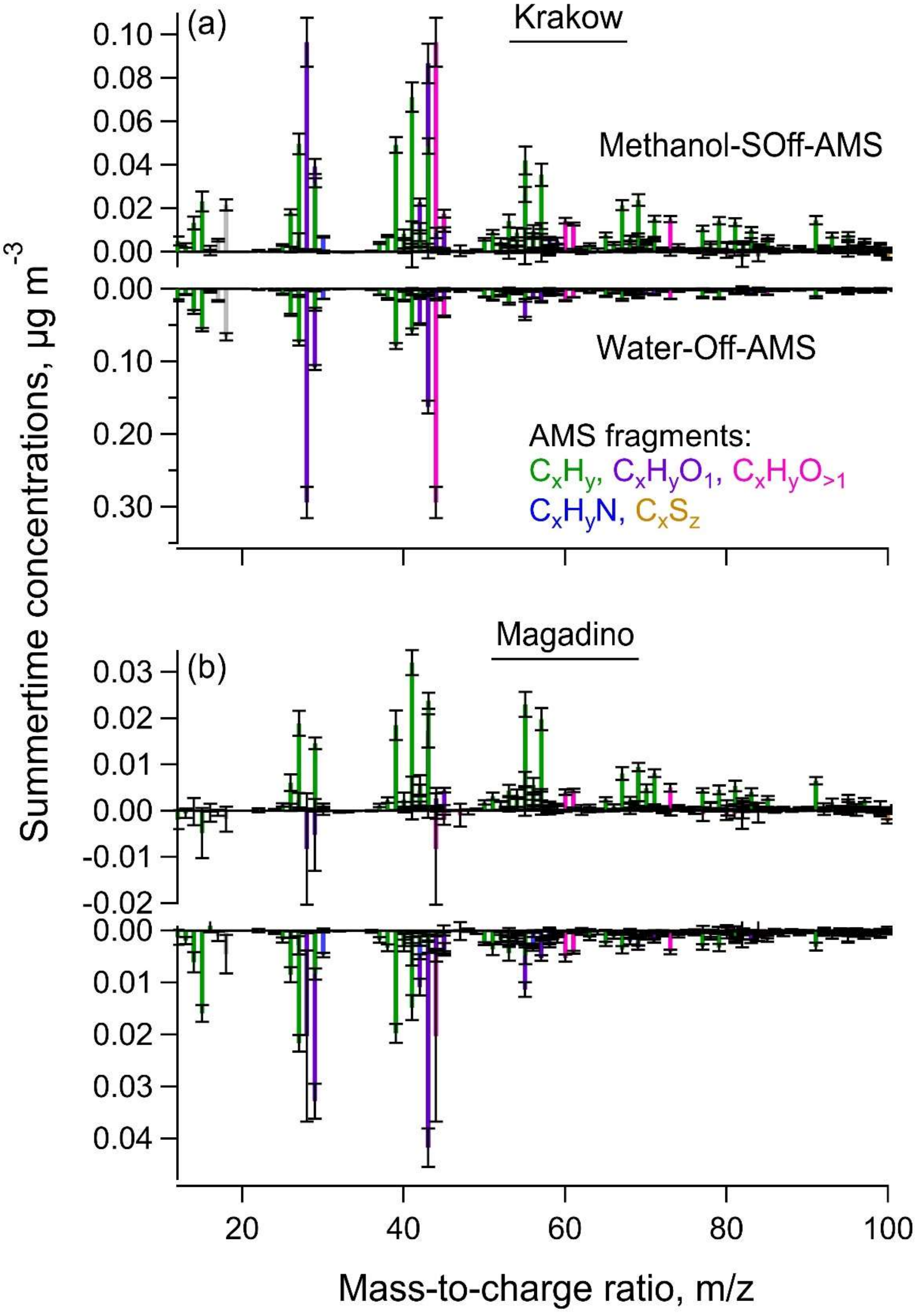

Figure S26. Summertime high-resolution mass spectra (including measurement uncertainties) of coarse OA from (a) Krakow and (b) Magadino extracted in methanol and water. CH_2O^+, CH_3O^+ and CH_4O^+ are blanked since those are solvent-related peaks.

Reference

1. Chen, G. *et al.* European aerosol phenomenology − 8: Harmonised source apportionment of organic aerosol using 22 Year-long ACSM/AMS datasets. *Environ Int* **166**, 107325 (2022).
